# Identification of natural compounds as SARS-CoV-2 inhibitors *via* molecular docking and molecular dynamic simulation

**DOI:** 10.3389/fmicb.2022.1095068

**Published:** 2023-02-01

**Authors:** Tiantian Han, Ziqing Luo, Lichun Ji, Peng Wu, Geng Li, Xiaohong Liu, Yanni Lai

**Affiliations:** ^1^The First Clinical Medical College, Guangzhou University of Chinese Medicine, Guangzhou, China; ^2^Laboratory Animal Center, Guangzhou University of Chinese Medicine, Guangzhou, China; ^3^The Third Clinical Medical College, Guangzhou University of Chinese Medicine, Guangzhou, China; ^4^School of Basic Medical Sciences, Guangzhou University of Chinese Medicine, Guangzhou, China

**Keywords:** molecular docking, molecular dynamics simulation, binding energy, SARS-CoV-2 inhibitors, Chinese herbal medicine

## Abstract

**Background:**

Base mutations increase the contagiousness and transmissibility of the Delta and Lambda strains and lead to the severity of the COVID-19 pandemic. Molecular docking and molecular dynamics (MD) simulations are frequently used for drug discovery and relocation. Small molecular compounds from Chinese herbs have an inhibitory effect on the virus. Therefore, this study used computational simulations to investigate the effects of small molecular compounds on the spike (S) protein and the binding between them and angiotensin-converting enzyme 2 (ACE2) receptors.

**Methods:**

In this study, molecular docking, MD simulation, and protein–protein analysis were used to explore the medicinal target inhibition of Chinese herbal medicinal plant chemicals on SARS-CoV-2. 12,978 phytochemicals were screened against S proteins of SARS-CoV-2 Lambda and Delta mutants.

**Results:**

Molecular docking showed that 65.61% and 65.28% of the compounds had the relatively stable binding ability to the S protein of Lambda and Delta mutants (docking score ≤ −6). The top five compounds with binding energy with Lambda and Delta mutants were clematichinenoside AR2 (−9.7), atratoglaucoside,b (−9.5), physalin b (−9.5), atratoglaucoside, a (−9.4), Ochnaflavone (−9.3) and neo-przewaquinone a (−10), Wikstrosin (−9.7), xilingsaponin A (−9.6), ardisianoside G (−9.6), and 23-epi-26-deoxyactein (−9.6), respectively. Four compounds (Casuarictin, Heterophylliin D, Protohypericin, and Glansrin B) could interact with S protein mutation sites of Lambda and Delta mutants, respectively, and MD simulation results showed that four plant chemicals and spike protein have good energy stable complex formation ability. In addition, protein–protein docking was carried out to evaluate the changes in ACE2 binding ability caused by the formation of four plant chemicals and S protein complexes. The analysis showed that the binding of four plant chemicals to the S protein could reduce the stability of the binding to ACE2, thereby reducing the replication ability of the virus.

**Conclusion:**

To sum up, the study concluded that four phytochemicals (Casuarictin, Heterophylliin D, Protohypericin, and Glansrin B) had significant effects on the binding sites of the SARS-CoV-2 S protein. This study needs further *in vitro* and *in vivo* experimental validation of these major phytochemicals to assess their potential anti-SARS-CoV-2.

**Graphical abstract:**
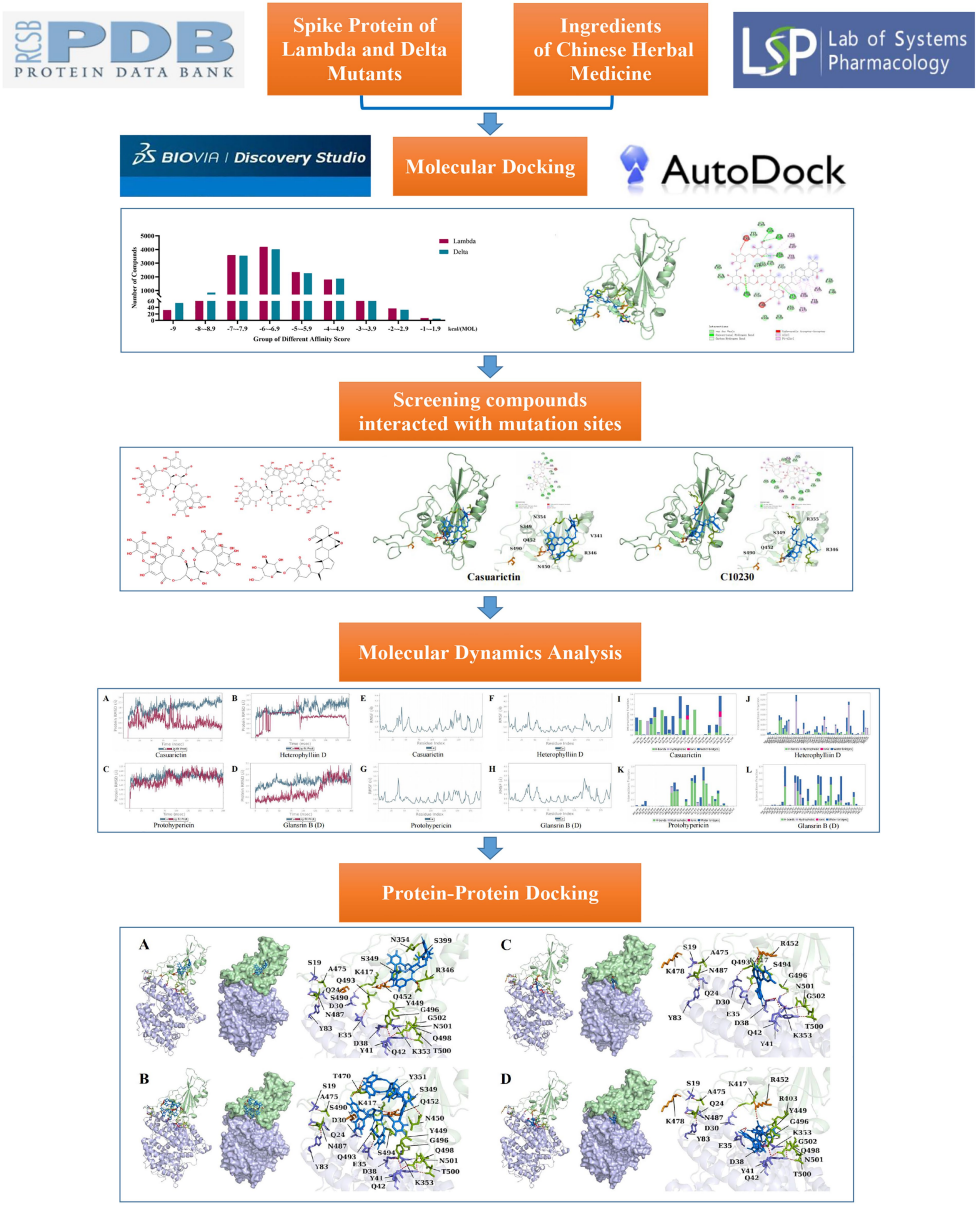

## 1. Introduction

COVID-19, caused by the Novel Coronavirus, remains a worldwide pandemic, with more than 628 million cumulative confirmed cases and more than 6.57 million cumulative deaths reported globally as of November 3, 2022. More and more studies have shown that COVID-19 can not only cause lung damage but also attack the liver (Bangash et al., 2020), kidneys (Balawender et al., 2022), heart (Madjid et al., 2020), reproductive system (Fan et al., 2020), and nervous system (Chuang et al., 2021). Patients infected with COVID-19 will show symptoms such as fever, exertion, dry cough, loss of smell and taste, dyspnea, and severely infected patients will have respiratory and circulatory failure and even multi-organ failure, which will lead to death (Tsang et al., 2021). In addition, the latest research shows that the long-term symptoms caused by the COVID-19 infection cause patients to develop muscle pain, tiredness, fear, depression, and other psychological disorders and increase the risk of cardiovascular disease (Hanson et al., 2022). Although coronaviruses have proofreading mechanisms to maintain their long genomes and have a relatively low mutation rate, different variants have emerged with severe economic and social impacts (Robson et al., 2020).

The SARS-CoV-2 virus infects *via* the engagement of human ACE2 by the virus receptor binding domain (RBD) of its S protein (Lan et al., 2020). The dominant strain currently circulating is the Omicron variant, the Delta variant, and the Lambda variant were the dominant strain worldwide before the Omicron variant, which mutations occurring in the RBD region (Pulliam et al., 2022). Delta has two mutations within its RBD, L452R, and T478K. The Lambda variant, once a major strain in Argentina and Chile, has two mutations in its RBD, L452Q, and F490S (Romero et al., 2021). Compared with the wild cohort, the Delta variant spreads faster, has a shorter incubation period, higher viral load, and longer viral clearance time, and elderly patients infected with Delta variety of concerns (VOCs) are more likely to develop critical illness (Wang et al., 2021). In addition to being highly transmissible, the Lambda variant is more infectious and resistant to neutralizing antibodies (NAb), resulting in a decrease in the protective effect of all currently approved anti-SARS-CoV-2 vaccines (Kimura and Kosugi et al., 2022). The Omicron variant is currently circulating; although the transmissibility and immune escape are both high, the pathogenicity has “substantially decreased.” Moreover, the probability of developing new crowns after infection with the Omicron variant has decreased by about 20%–50% compared with the Delta variant (Nyberg et al., 2022; Xie et al., 2022). At present, although vaccines can provide a high level of prevention of hospitalization and reduce mortality, vaccines cannot prevent new coronavirus infection or reinfection, and there are currently no effective anti-new coronavirus drugs (Nyberg et al., 2022).

Studies have shown that traditional Chinese medicine has played a particular role in the treatment of COVID-19, suggesting that antiviral and even anti-variant drug candidates can be found in various Chinese herbal medicines (Ren et al., 2020; Runfeng et al., 2020). Drug screening in the preclinical and clinical stages is costly and time-consuming (Walls et al., 2020). Virtual screening by computer can provide rapid, considerable, and new testable hypotheses for drug repositioning (Cheng et al., 2017).

In this study, the protein structures of Delta and Lambda variants were constructed and, respectively, docked with 12,978 small molecule compounds, which were verified and extracted from Chinese herbal medicine downloaded from the Traditional Chinese Medicine Systems Pharmacology Database and Analysis Platform (TCMSP). The compound with the highest docking score was screened out, and then MD was simulated. The protein–protein docking was carried out to understand its mode of action. This study is expected to provide a reference for screening anti-virus and even anti-mutant strains.

## 2. Materials and methods

### 2.1. Protein structure and evaluation

Download the S protein and ACE2 binding structure file (6LZG) from the PDB database.^1^ The mutation sites of the Delta and Lambda strain of the binding domain of the S protein were confirmed. Using PyMOL 2.1 to carry out virtual mutation function, 452 amino acids of S protein RBD were mutated from leucine (Leu, L) to glutamine (Gln, Q) and 490 amino acids were mutated from Fnylalanine (F, F) to serine (Ser, S) to form Lambda strain. The 452 amino acid of RBD of S protein was mutated from L to arginine (Arg, R), and the 478 amino acid was mutated from threonine (Thr, T) to lysine (Lys, K) to form Delta strain. The protein was added hydrogen by Autodcok Tool 1.5.6 and formed a PDBQT file. The rationality of protein conformations was evaluated by Ramachandran plot using UCLA-DOE’s SAVES server v 6.0.^2^

### 2.2. Compound structure and optimization

The three-dimensional conformations of the 13,144 small molecule compounds from 500 Chinese herbs were downloaded from the TCMSP database.^3^ The compounds were minimized by assigning force field MM2 by using ChemBio3D Ultra 13.0, and the optimized structure was prepared.

### 2.3. Virtual screening and molecular docking

Autodock Vina 1.1.2 was used in silicon docking. The docking was carried out, and the binding pocket covered the mutation base with the following parameters −42.602, 33.01, and 9.399 for the X, Y, and Z axes, respectively. These coordinates represent the binding site area covering the C-terminal domain (CTD) of the Sprotein of SARS-CoV-2. The x, y, and z length of the grid box is 50. The affinity score was analyzed by sectional statistics, and GraphPad Prism 9.0 was used for visualization.

### 2.4. Molecular dynamics simulation

MD simulations of protein and compound complexes were performed by Desmond v2020. OPLS3e was selected as the molecular field for MD simulation.TIP3 water model was used in the MD system. Neutralize the system charge by adding ions. The energy minimization of the entire system is achieved by using the OPLS3e force field since it is a full atomic-type force field. The geometry of water molecules, bond lengths, and bond angles of heavy atoms are constrained by the SHAKE algorithm. A continuous system is simulated by applying periodic boundary conditions. Long-range static electricity is maintained by the particle mesh Ewald method. An NPT method harness at 300 K and 1.0 bar was used to balance the system. Berendsen coupling algorithm is used to couple temperature–pressure parameters. At a later stage of preparation of the system, 100 ns was run at a time step of 1.2 fs. The track was recorded every 100 ps, recording a total of 1,000 frames. The Root Mean Square Deviation (RMSD) of backbone atoms was calculated, and a graphical analysis was performed to understand the nature of protein-ligand interactions. The Root Mean Square Fluctuation (RMSF) of each residue was calculated to understand the major conformational changes of the residue between the initial state and the kinetic state.

### 2.5. Protein–protein docking

Protein structure (6LZG) was obtained from the PDB database (see text footnote 1, respectively) and processed by PyMOL 2.1 software, including the removal of water and ions, protonation, the addition of missing atoms, and completion of chemical groups. HDOCK SERVER^4^ is used to set the protein as a rigid state. The range of Receptor protein was set as 19:A, 24:A, 35:A, 38:A, 42:A, 353:A, and 30:A. The range of Delta and Lambda S protein was set as 487:B, 475:B, 417:B, 493:B, 502:B, 496:B, 449:B, and 498:B. 100 conformations were generated in silicon docking, and the best conformation was selected by scoring in ascending order.

## 3. Results

### 3.1. Evaluation of protein structure rationality

**Figure 1:**
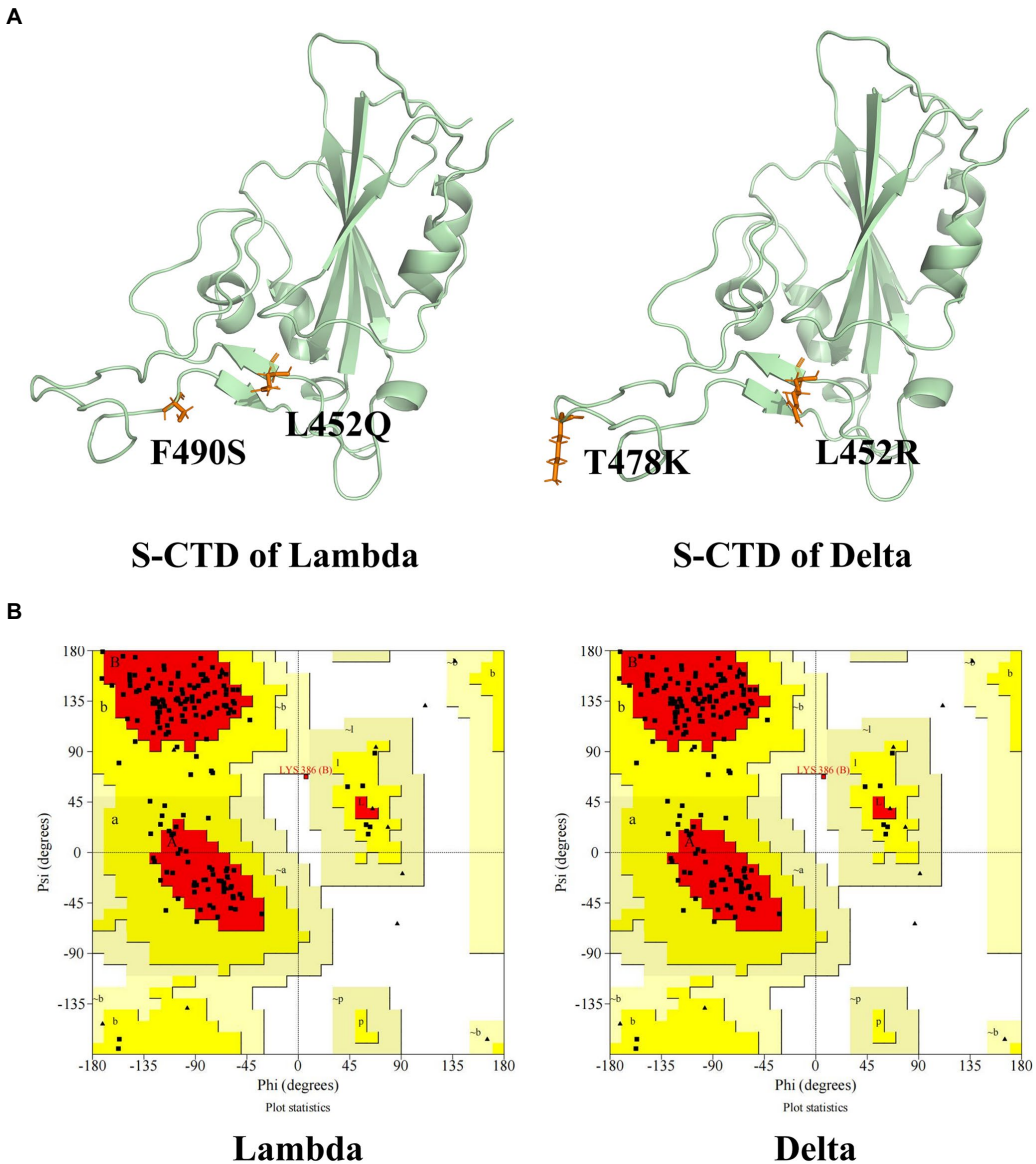
The CTD of S protein structure and evaluation. **(A)** The tertiary structure of CTD of S protein of Lambda and Delta strain. **(B)** The Ramachandran plot of the mutation S protein. The mutation residues were shown in orange and CTD of S protein was shown in palegreen.

Ramachandran Plot was used to assess the rationality of the mutation S protein (Morris et al., 1992). As can be seen in Figure 1, the total number of residues of CTD of S protein is 195. The residues in most favored regions take part of 82.1%, and residues in additional allowed regions were 17.3%. The residues in disallowed regions were 0.6% which was <5%, indicating the rationality of the structure of the S protein of the mutation strain.

### 3.2. Optimization results of compound structure

There were 13,144 compounds in the TCMSP database, and 12,978 compounds were optimized after removing the compounds with unreasonable structures 166 compounds could not be optimized.

### 3.3. Molecular docking analysis

Affinity score was used to evaluate the result of virtual screening. The lower the affinity score, the more stable the confirmation. The docking results of 12,978 compounds with Lambda S-CTD and Delta S-CTD were ranked from lowest to highest according to affinity scores.

**Figure 2:**
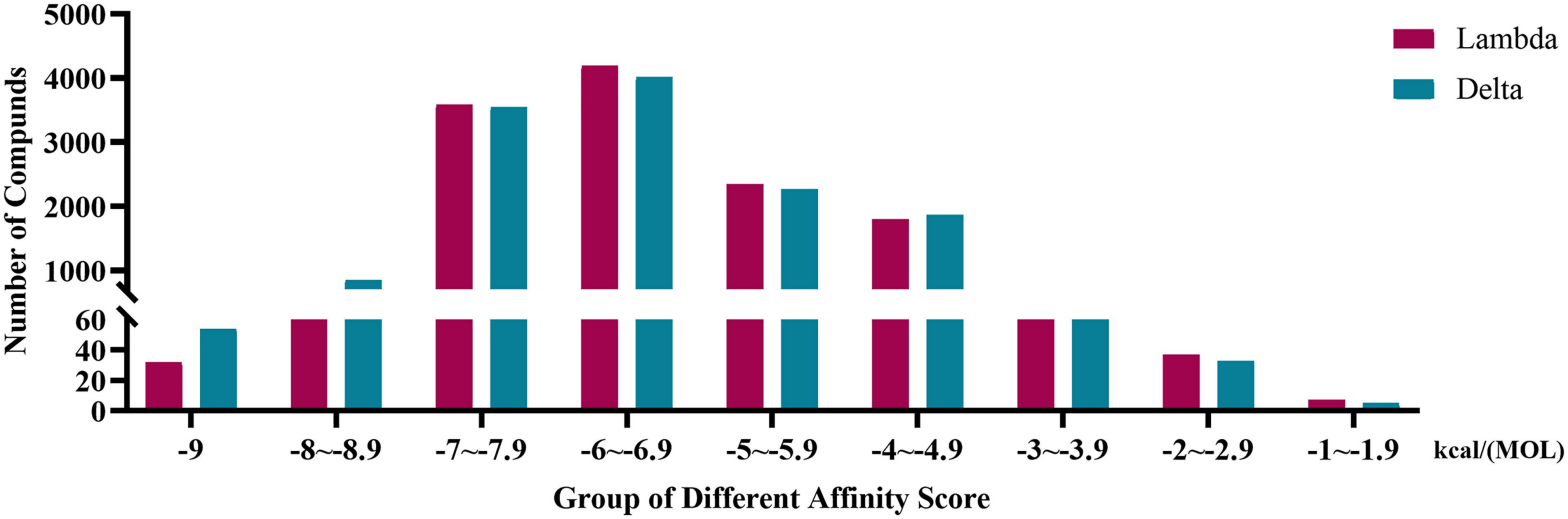
Distribution bar graph of the affinity score of small molecular compounds with Lambda and Delta strain.

The affinity scores were normally distributed. Most compounds were concentrated in a fraction of −4 to −7.9 kcal/(MOL). Generally, the conformations with affinity scores below −8 were considered reliable. There were 734 (5.66%) Lambda conformation and 910 (7.01%) Delta confirmations below −8 (Figure 2), indicating those compounds which consisted of conformation had an interactive relationship with CTD of S protein.

The top 20 compounds were shown in Tables 1, 2. Among the top 100 results, not all compounds bind to the mutation base. By checking the 9 conformations manually of the top 100 results, the conformation of compounds bound to the mutation base was selected and listed in Tables 3, 4 according to affinity score from lowest to highest. The 2D structure of compounds was shown in Tables 3, 4.

**Table 1:** Molecular docking results of Lambda S-CTD and compounds (top 20).

Rank	Affinity (kcal/MOL)	MOL_ID	molecule_name
1	−9.7	MOL000753	clematichinenoside AR2
2	−9.5	MOL006876	atratoglaucoside,b
3	−9.5	MOL007238	physalin b
4	−9.4	MOL006874	atratoglaucoside,a
5	−9.3	MOL003009	Ochnaflavone
6	−9.3	MOL003351	cyclopseudo-hypericin
7	−9.3	MOL005678	periplocoside J
8	−9.3	MOL006757	Bryonolic acid
9	−9.3	MOL007353	solamargine
10	−9.2	MOL003278	salaspermic acid
11	−9.2	MOL005671	periplocoside C
12	−9.2	MOL009468	β1-solamargine
13	−9.2	MOL009472	26-O-β-D-glucopyranosyl-nuatigenin-3-O-α-L-rhamnopyranosyl(1→2)-β-D-glucopyranoside
14	−9.2	MOL011100	bisindigotin
15	−9.1	MOL002067	hypericin
16	−9.1	MOL002659	kihadanin A
17	−9.1	MOL004500	Markogenin-3-O-beta-D-glucopyranosyl-(1–2)-beta-D-galactopyranoside
18	−9.1	MOL005459	Diosgenin-3-O-beta-D-glucopyranoside
19	−9.1	MOL006855	solamargine
20	−9.1	MOL006895	glaucoside,d

**Table 2:** Molecular docking results of Delta S-CTD and compounds (top 20).

Rank	Affinity (kcal/MOL)	MOL_ID	molecule_name
1	−10	MOL007062	neo-przewaquinone a
2	−9.7	MOL011124	Wikstrosin
3	−9.6	MOL004521	xilingsaponin A
4	−9.6	MOL011030	ardisianoside G
5	−9.6	MOL011990	23-epi-26-deoxyactein
6	−9.6	MOL012727	mulberrofuran K
7	−9.5	MOL004509	Timosaponin A III
8	−9.5	MOL007238	physalin b
9	−9.5	MOL009474	26-O-β-D-glucopyranosylnuatigenin-3-O-α-L-rhamnopyranosyl(1→2)-o-[β-D-glucopyranosyl(1→4)]-β-D-glucopyranoside
10	−9.4	MOL004444	Ziebeimine
11	−9.4	MOL005626	cynapanoside B
12	−9.4	MOL006757	Bryonolic acid
13	−9.4	MOL006874	atratoglaucoside,a
14	−9.4	MOL006876	atratoglaucoside,b
15	−9.4	MOL011540	withanolide D
16	−9.4	MOL012929	glaucoside C
17	−9.3	MOL003351	cyclopseudo-hypericin
18	−9.3	MOL005678	periplocoside J
19	−9.3	MOL008582	trillin
20	−9.3	MOL011486	Datuarmeteloside B

**Table 3:** Molecular docking results of Lambda S-CTD mutant bases and compounds.

Rank	Affinity (kcal/MOL)	MOL_ID	Molecule name	Structure
1	−9.1	MOL010726	Casuarictin	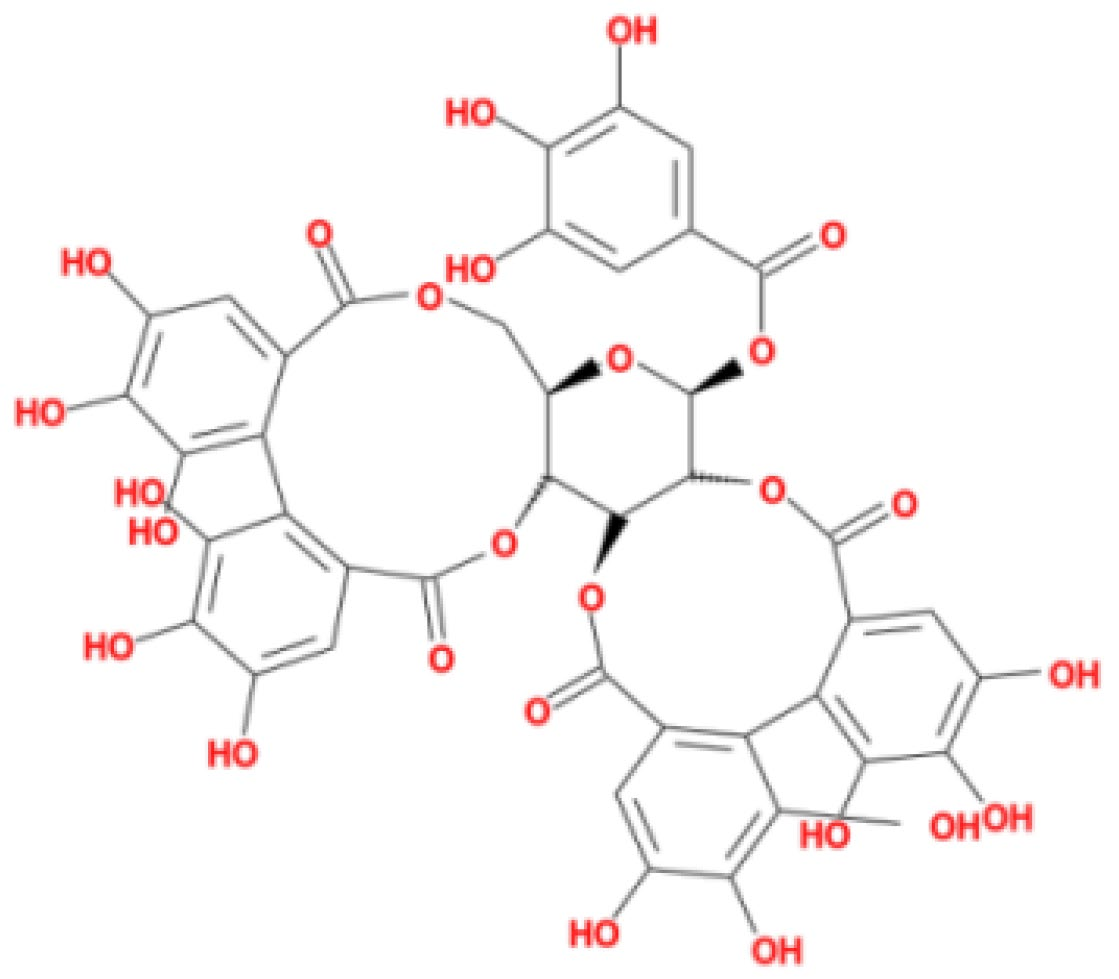
2	−9.0	MOL002505	C10230	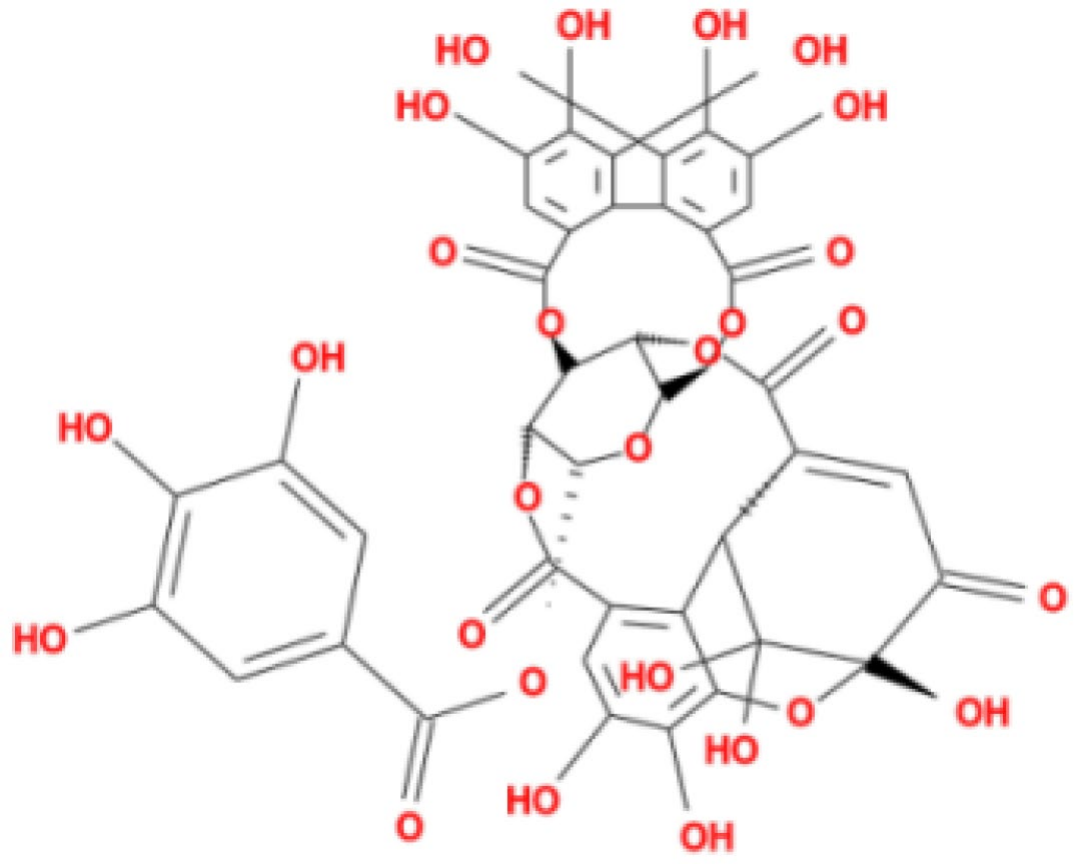
3	−8.9	MOL005678	Periplocoside J	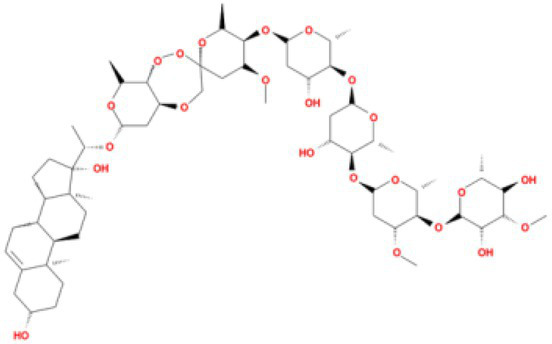
4	−8.1	MOL009110	Heterophylliin D	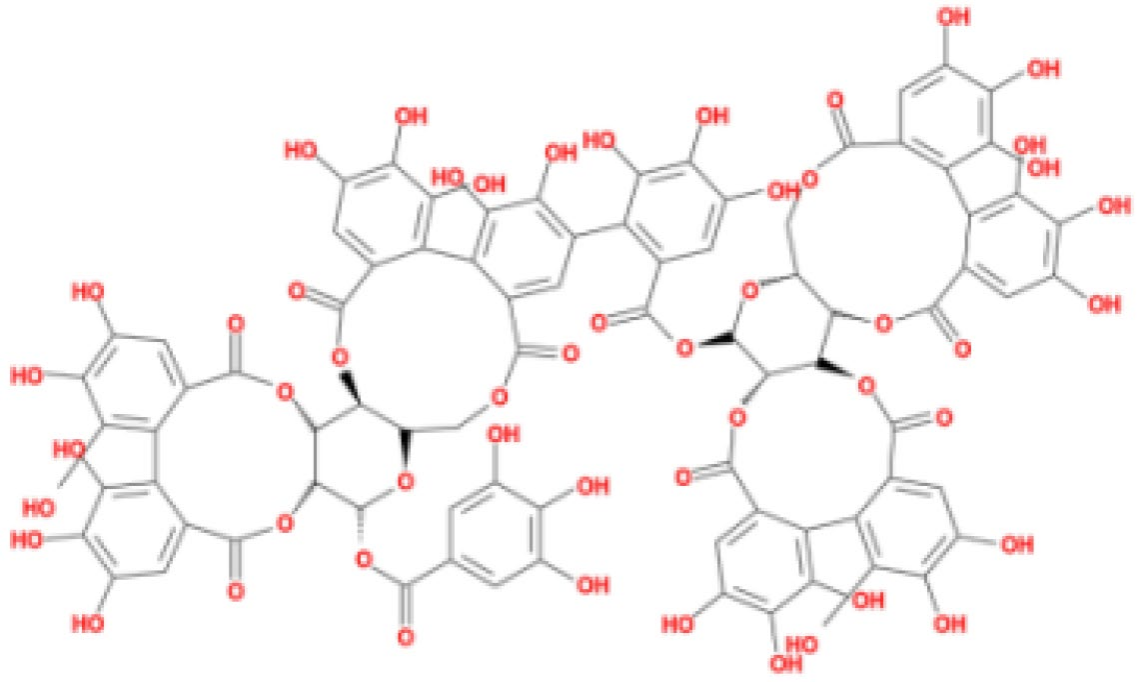
5	−7.9	MOL003351	Cyclopseudo-hypericin	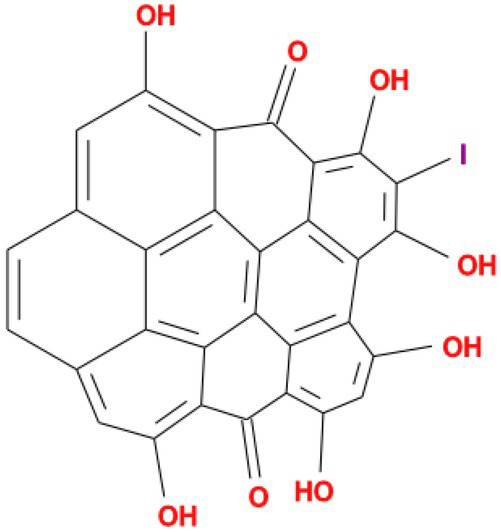
6	−7.9	MOL006895	Glaucoside,d	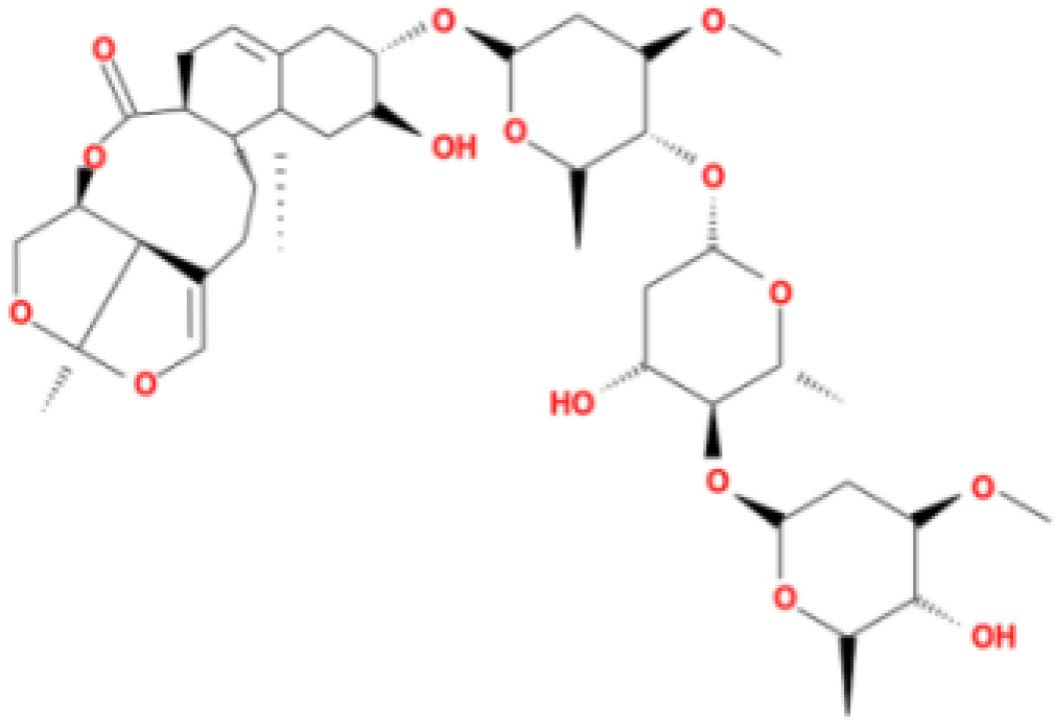
7	−7.8	MOL002067	Hypericin	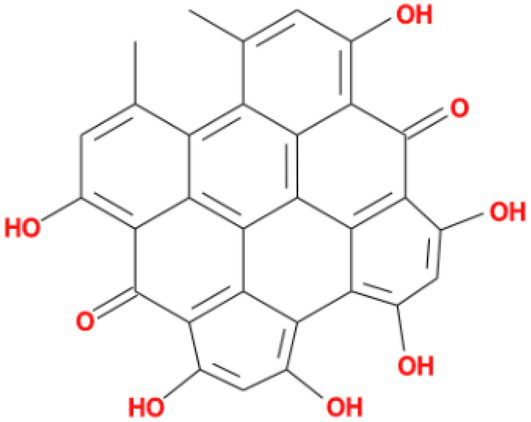
8	−7.6	MOL009468	β1-solamargine	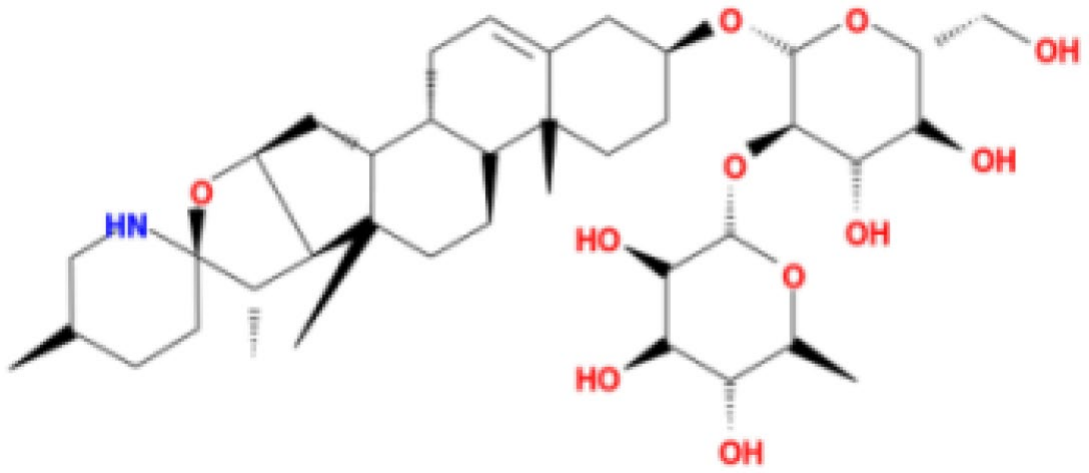
9	−7.5	MOL012732	Mulberrofuran Q	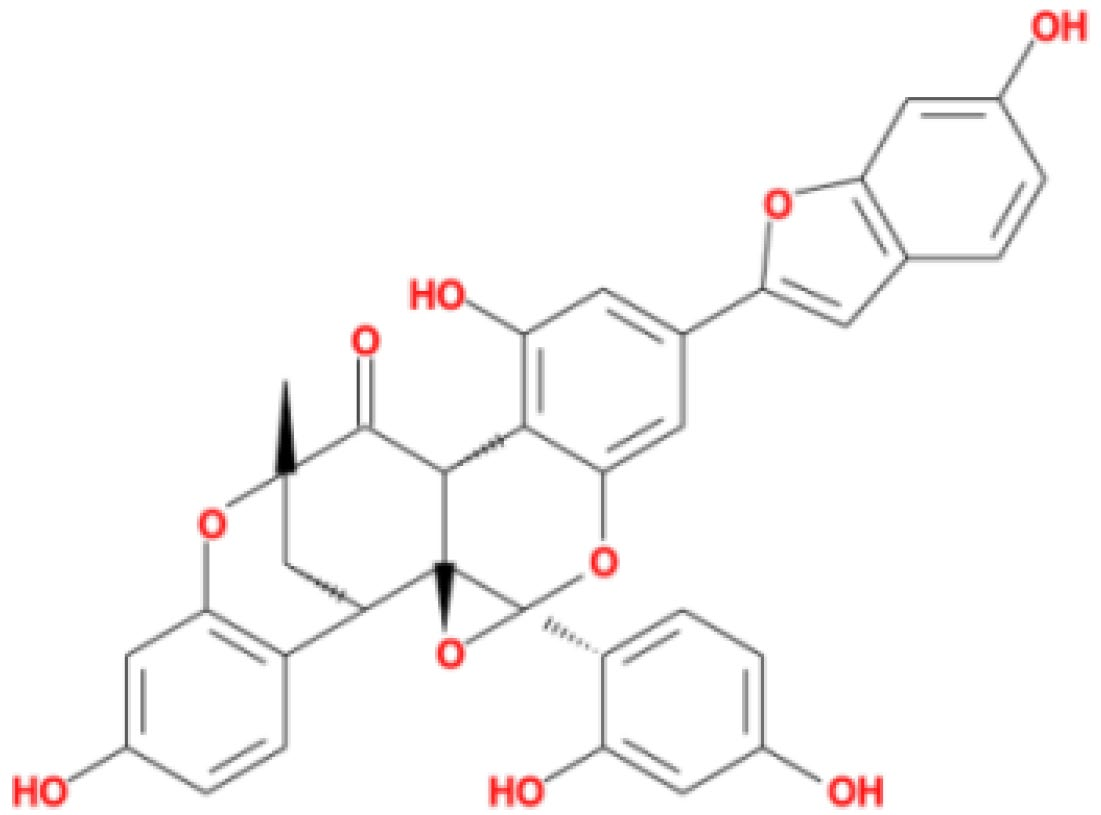
10	−7.2	MOL006876	Atratoglaucoside,b	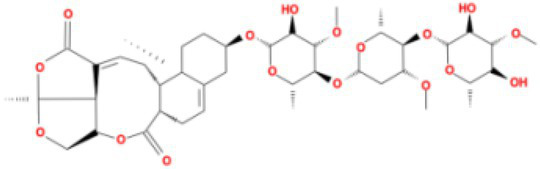

**Table 4:** Molecular docking results of Delta S-CTD mutant bases and compounds.

Rank	Affinity (kcal/MOL)	MOL_ID	Molecule name	Structure
1	−8.9	MOL009107	Glansrin B	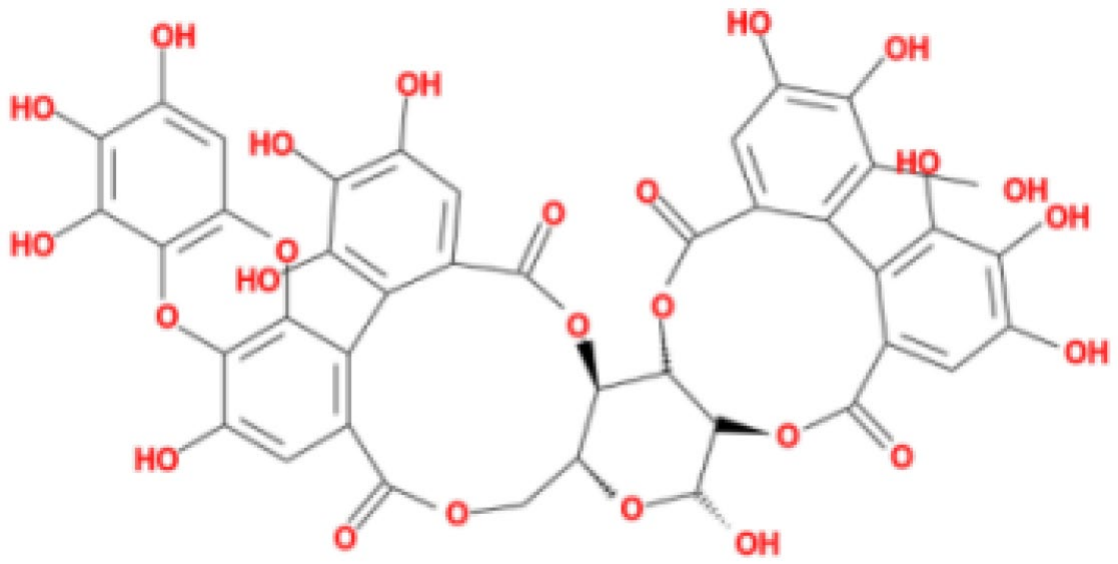
2	−8.9	MOL008305	Ardisiacrispin A	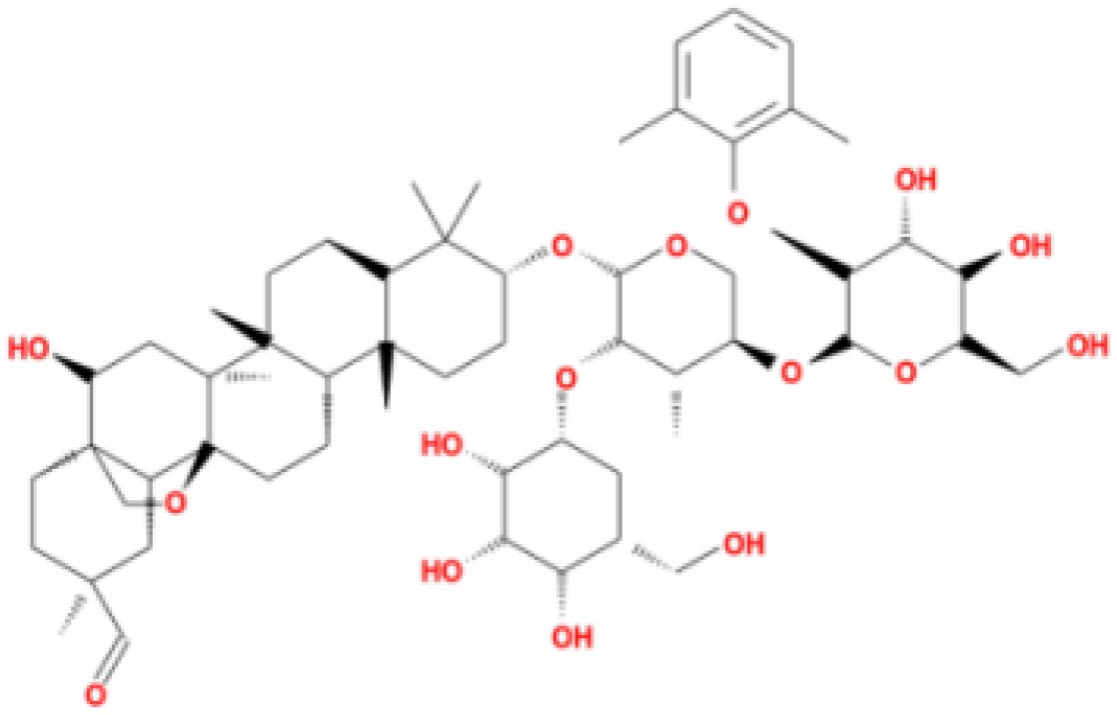
3	−8.6	MOL005678	Periplocoside J	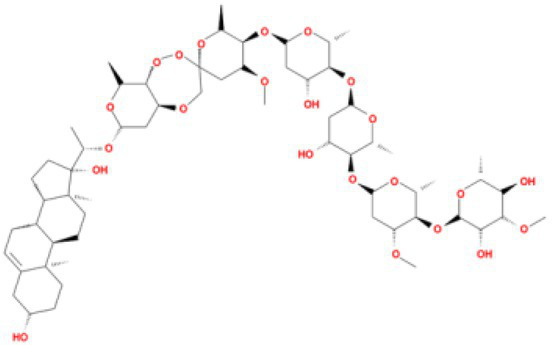
4	−8.4	MOL003351	Cyclopseudo-hypericin	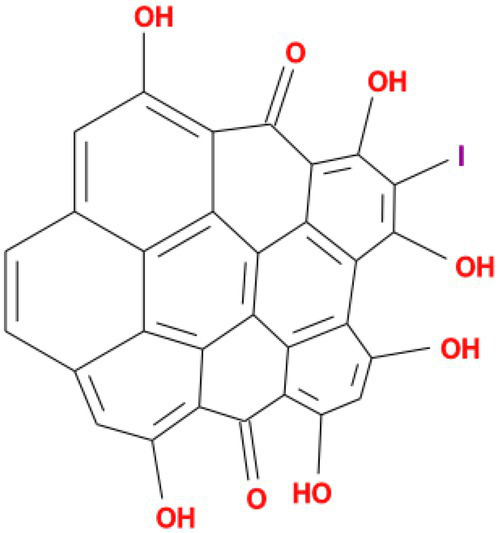
5	−8.4	MOL002037	Amentoflavone	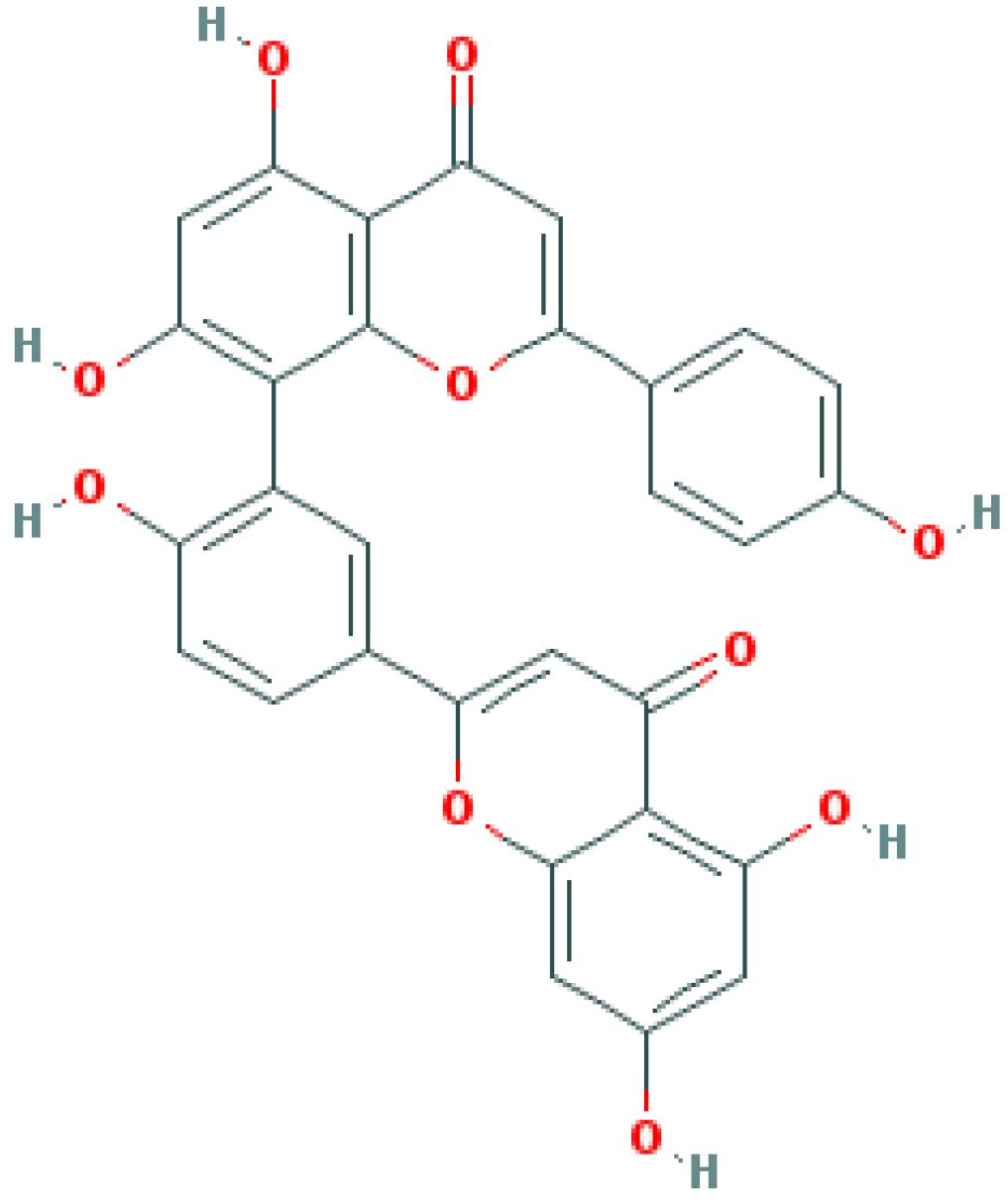
6	−8.4	MOL003009	Ochnaflavone	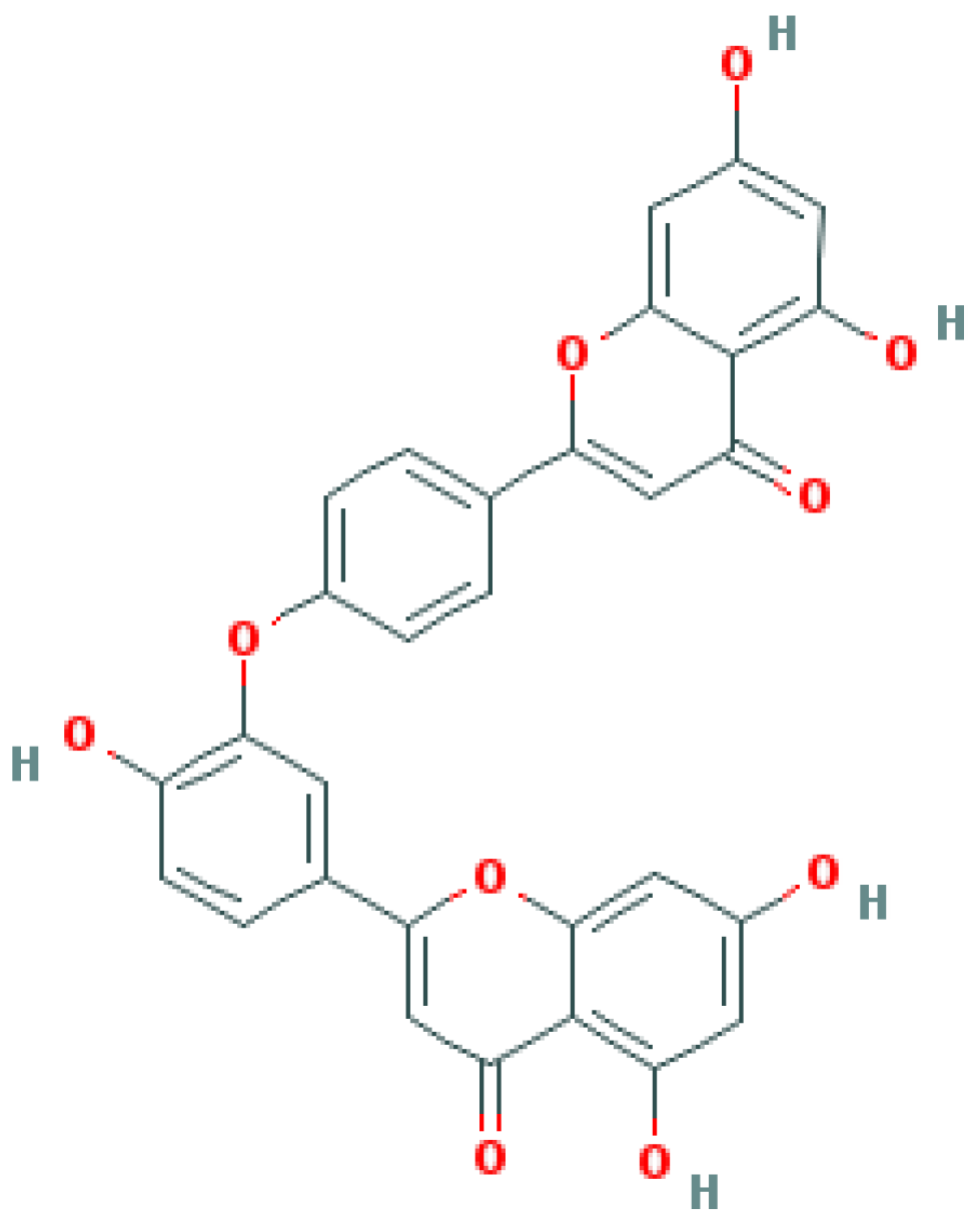
7	−8.3	MOL002067	Hypericin	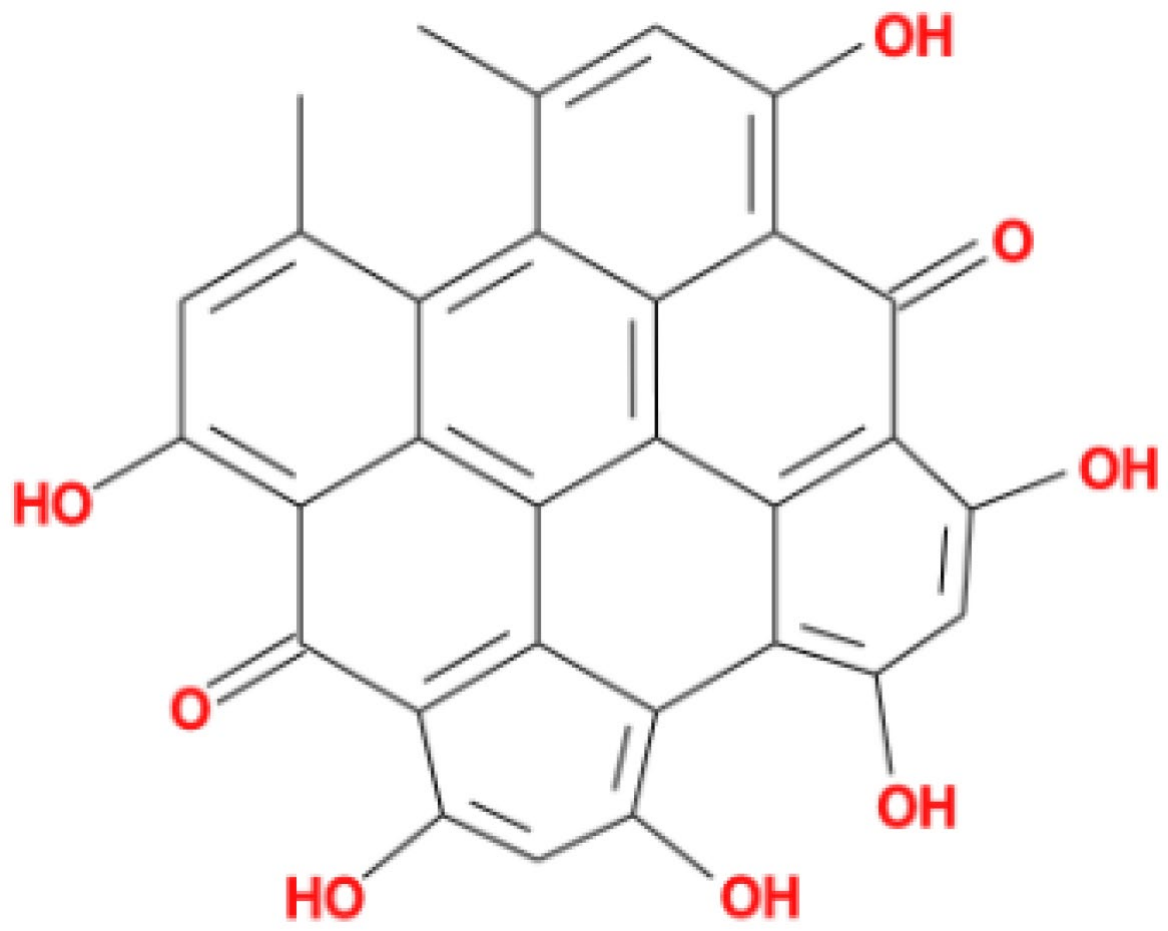
8	−8.2	MOL003350	Protohypericin	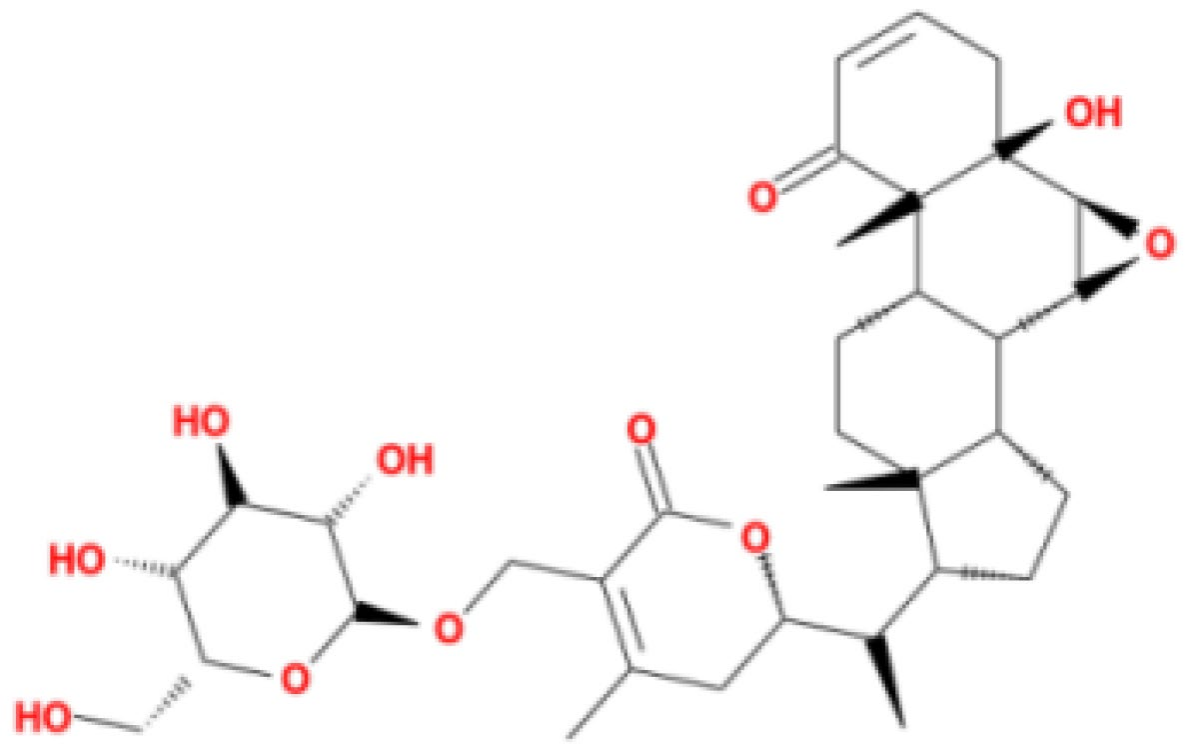
9	−8.2	MOL011486	Datuarmeteloside B	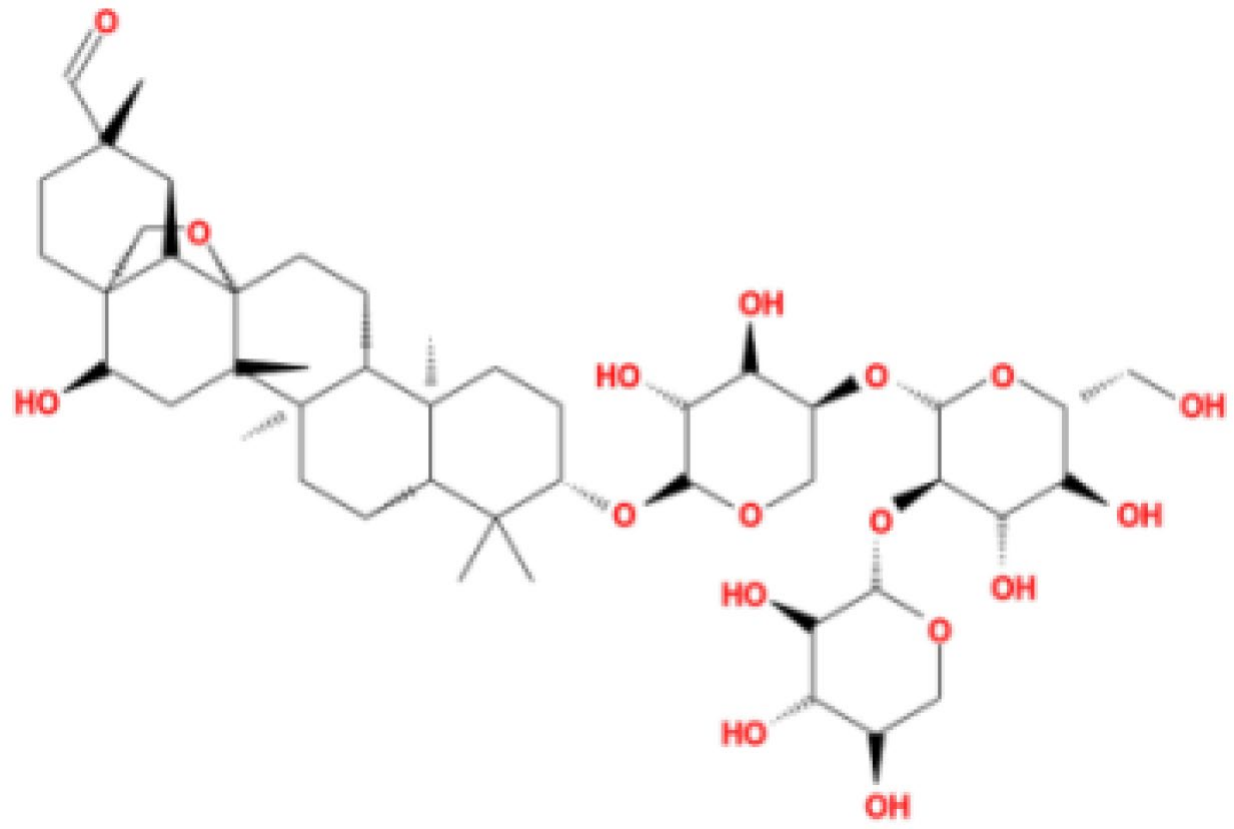
10	−8.2	MOL010968	Primulanin	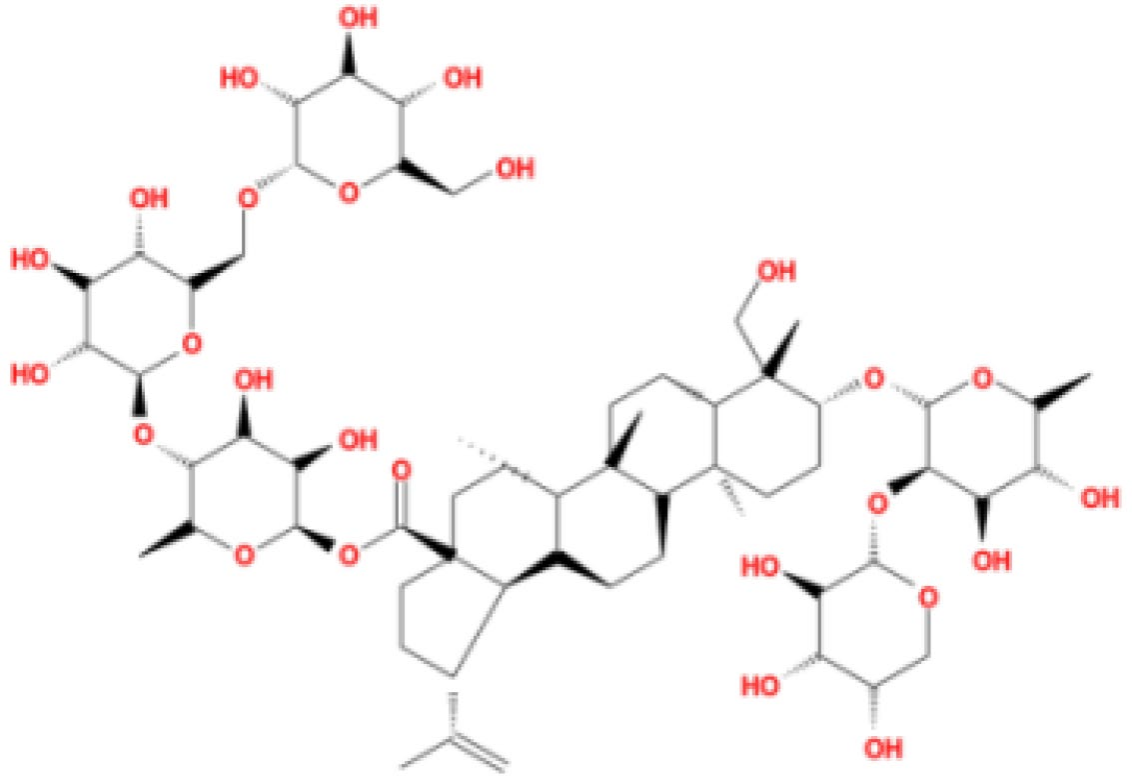

**Figure 3:**
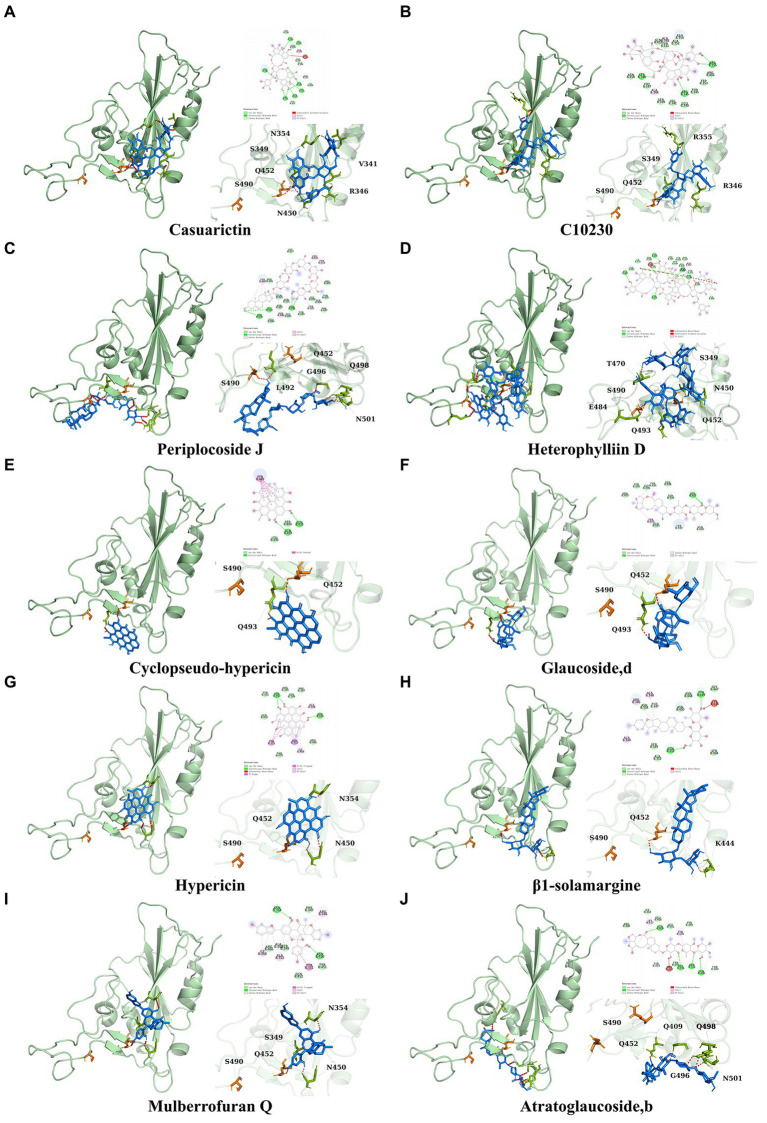
The top 10 conformations of the small molecule compounds bind to the Lambda strain mutation base. **(A)** Casuarictin. **(B)** C10230. **(C)** Periplocoside J. **(D)** Heterophylliin D. **(E)** Cyclopseudo-hypericin. **(F)** Glaucoside, d. **(G)** Hypericin. **(H)** β1-solamargine. **(I)** Mulberrofuran Q. **(J)** Atratoglaucoside, b.

**Figure 4:**
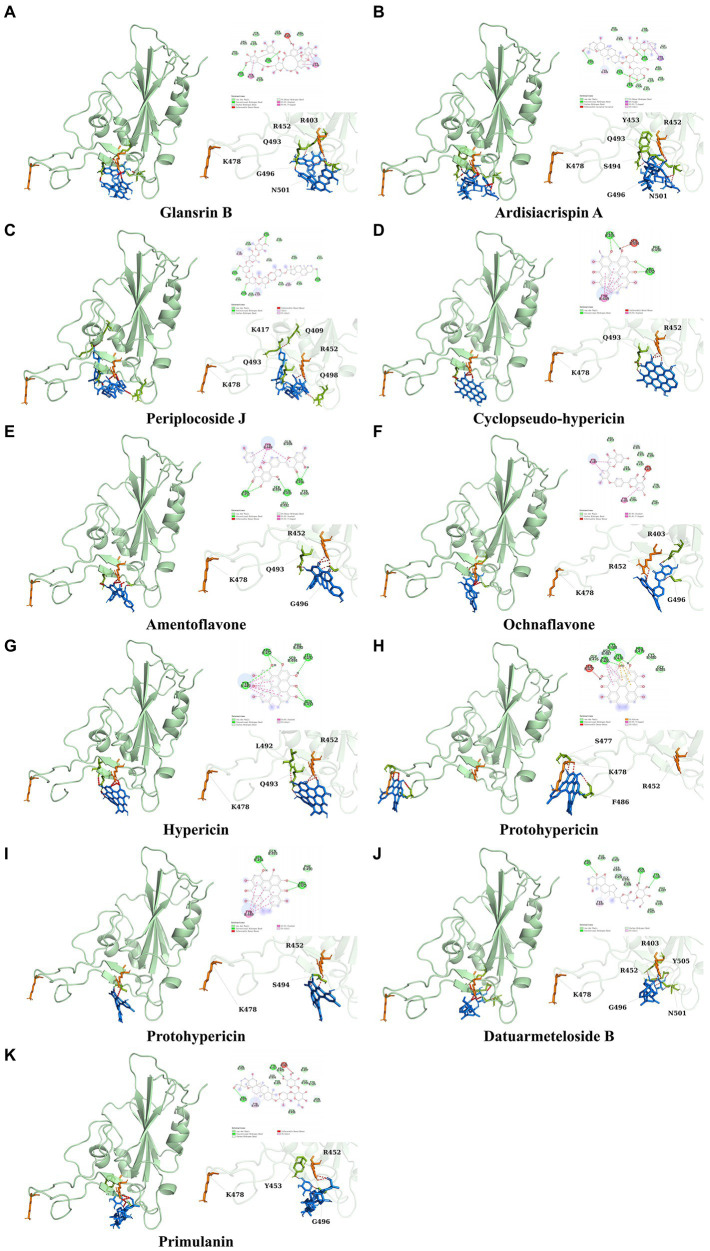
The top 10 conformations of the small molecule compounds bind to the Delta strain mutation base. **(A)** Glansrin B. **(B)** Ardisiacrispin A. **(C)** Periplocoside J. **(D)** Cyclopseudo-hypericin. **(E)** Amentoflavone. **(F)** Ochnaflavone. **(G)** Hypericin. **(H, I)** Protohypericin. **(J)** Datuarmeteloside B. **(K)** Primulanin.

It showed that Casuarictin, C10230, Cyclopseudo-hypericin, Glaucoside,d, Hypericin, β1-solamargine, Mulberrofuran Q, and Atratoglaucoside, bind to Q452 of lambda strain, while Periplocoside J binds to S490. Heterophylliin D binds to both Q452 and S490 through van der weals and conventional hydrogen bonds (Figure 3, Table 5). Glansrin B, Ardisiacrispin A, Periplocoside J, Cyclopseudo-hypericin, Amentoflavone, Ochnaflavone, Hypericin, Datuarmeteloside B, and Primulanin only bind to R452 of delta strain, while Protohypericin binds to R452 and K478, respectively, in a different conformation. Protohypericin binds to R452 through conventional hydrogen bond, and it binds to K478 through Pi-cation, Pi-Alkyl and conventional hydrogen bond (Figure 4, Table 6).

**Table 5:** The residues for the chemical bonds between compounds and spike proteins of Lambda strain.

Compounds	Van de waals	Conventional hydrogen bond	Unfavorable acceptor-acceptor	Carbon hydrogen bond	Pi-Alky1
Casuarictin	F357, Y451, N354, K356, I468	E340, V341, R346, S349, Y351, N450, Q452	S399	A348	A344, A352
Heterophylliin D	P491, E471, I472, S490, S494, Y451, Y449, F347, A348, S349, Y351, S469, I468	Q493, Q452, E484, T470	L492	N450	A352

**Table 6:** The residues for the chemical bonds between compounds and spike proteins of Delta strain.

Compounds	Van de waals	Conventional hydrogen bond	Carbon hydrogen bond	Unfavorable donor-donor	Pi-Pi T-shaped	Pi-Pi Stacked
Glansrin B	Y453, Y495, F497, S494, R452, Q498	G496, N501	R403	Q493	Y505	Y449
Protohypericin (Conformation 1)	Q476, N487, C489, G485	P479, C488, K478, F486	–	S477	–	Y449
Protohypericin (Conformation 2)	Q493, F490	S494, R452	–	–	–	Y449

### 3.4. Molecular dynamics analysis

Molecular docking cannot fully consider the flexibility of protein structure. To further elucidate the critical interactions between small molecule compounds and S protein, MD simulation was performed on the optimal molecular docking model in this study. The model with the highest score and the model with the most mutation sites were considered to be the best model. They were Casuarictin, Heterophylliin D and S protein of Lambda strain, Protohypericin, and Glansrin B and S protein of Delta strain, respectively.

**Figure 5:**
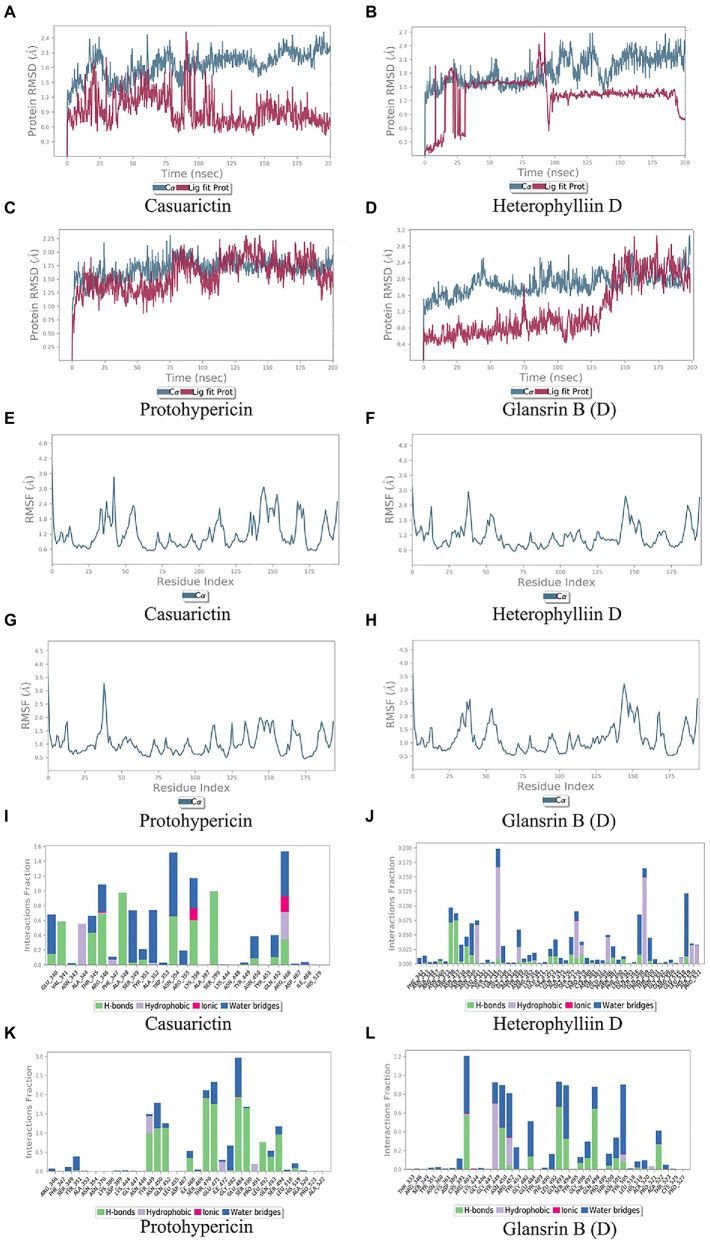
Molecular dynamics analysis of the top 2 conformations of small molecular compounds bind to mutation base. The results of protein RMSD, the RMSF of residue index and interactions fraction of Casuarictin **(A, E, I)**, Heterophylliin D **(B, F, J)** Protohypericin **(C, G, K)** and Glansrin B **(D, H, L)** binds to the S-CTD of Lambda strain and Delta strain.

In MD trajectory analysis, root means square deviation (RMSD) and root mean square fluctuation (RMSF) is the most frequently used index. The larger RMSD is, the more unstable the conformation is. As seen from Figures 5A–D, the small molecule fluctuated at the beginning and gradually tend to be stable in the movement process. In the MD simulation of 200 ns, the RMSD between the four compounds and the S protein reached a relative equilibrium state. It revealed that these four compounds approach the appropriate position of the S protein, thus promoting the stability between them and indicating that the small molecule was well combined with the protein surface. RMSF can be used to observe the allosteric of local sites in the simulation process. The larger the RMSF is, the more pronounced the conformational change of residue is. 0.2 was used to be the cut-off value. As shown in Figures 5E–H, in the binding between Casuarictin and S protein, residues 30–40, 140–145, 150–156 were Highly fluctuating. In the binding between Heterophylliin D and S protein, residues 35–40 and 140–145 were highly fluctuating. In the binding between Protohypericin and S protein, residues 35–40 were highly fluctuating, while the residues 145–150 were highly fluctuating in the binding between Glansrin B and S protein.

Protein-ligand interactions can be monitored throughout the simulation. These interactions can be classified and summarized by type, as shown in Figures 5I–L. Protein-ligand interactions was divided into four types. They were hydrogen bonds, hydrophobic interaction, ions, and water bridges. The binding of Casuarictin with S of Lambda strain and the binding of Protohypericin and Glansrin B with S-CTD of Delta strain were mainly connected with H-bonds. The binding of Heterophylliin D with S of Lambda strain displayed with hydrophobic interaction.

Combing the result of RMSD, RMSF, and Protein-ligand interactions, Casuarictin and Heterophylliin D bind to the S-CTD of the Lambda strain and showed reliable stability. Protohypericin and Glansrin B bind to S-CTD of Delta strain and displayed reliable stability.

### 3.5. Protein–protein docking analysis

ACE2 receptors of the host provide a binding site for the S protein of SARS-CoV-2, which indicates that the destruction of the binding between these two proteins provides the potentiality for COVID-19 treatment. To explore the effect of compound candidates during the recognition and fusion between S protein and ACE2, protein–protein docking was carried out. Binding Energy (kcal/mol) was used to indicate the stability between two proteins. The lower the score, the stronger the bond. As shown in Table 7, the predicted binding energy of the Lambda strain and Delta strain were −349 kcal/mol and −355 kcal/mol, respectively, which were higher than the standard strain.

**Table 7:** The results of protein–protein docking.

	Binding Energy (kcal/mol)	The residue of ACE2	The residue of S protein
S protein of Standard strain with ACE2	−330	S19, Q24, D30, E35, D38, Y41, Q42, Y83, K353	K417, Y449, A475, N487, Q493, G496, Q498, T500, N501, G502
S protein of Lambda strain with ACE2	−349	S19, Q24, D30, E35, Q42, K353	A475, N487, Q493, K417, G496, G446, G502, Q498
Casuarictin binding to S protein of Lambda strain with ACE2	−329	S19, Q24, D30, E35, D38, Y41, Q42, Y83, K353	K417, Y449, A475, N487, Q493, G496, Q498, T500, N501, G502
Heterophylliin D binding to S protein of Lambda strain with ACE2	−344	S19, Q24, D30, E35, D38, Y41, Q42, Y83, K353	K417, Y449, A475, N487, Q493, G496, Q498, T500, N501
S protein of Delta strain with ACE2	−355	S19, Y83, K31, K353, N330	A475, N487, L492, Q493, G496, G446, G502, Q498, T500
Glansrin B binding to S protein of Delta strain with ACE2	−143	S19, Q24, D30, E35, D38, Y41, Q42, Y83, K353	K417, A475, N487, R403, Q393, R452, Y449, G496, Q498, T500, N501, G502
Protohypericin binding to S protein of Delta strain with ACE2	−340	S19, Q24, D30, E35, D38, Y41, Q42, Y83, K353	K417, R452, A475, N487, Q493, S494, G496, T500, N501, G502

**Figure 6:**
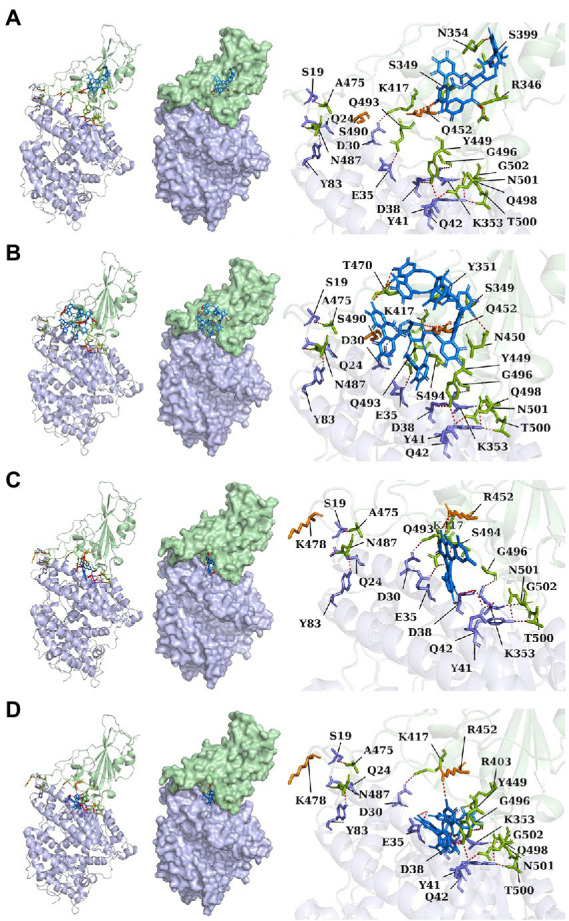
The cartoon, surface, and stick of Casuarictin **(A)**, Heterophylliin D **(B)**, Protohypericin **(C)**, and Glansrin B **(D)** binding to S-CTD dock with ACE2 receptor. The small molecule compounds were shown in marine. The S-CTD of lambda and delta was shown in pale green. The residue of S-CTD was shown in split-pea. The ACE2 receptor was shown in light blue. The residue of the ACE2 receptor was shown in the slate. The hydrogen bonds were shown in red.

Lambda and Delta strains are more contagious than standard strains, as seen clinically, and reflected in the binding energy of protein–protein docking results mentioned above. However, when the S protein binds to small molecule compounds, such as Casuarictin, Heterophylliin D, Protohypericin, and Glansrin B, the binding energy between the S protein and ACE2 receptor was raised, and the stability was shaken. The binding energy between the S protein of Lambda-strain along with Casuarictin and Heterophylliin D were −329 kcal/mol and −344 kcal/mol, which were higher than −349 kcal/mol of S protein binding to ACE2 alone. The binding energy of Glansrin B, along with S protein and ACE2, was significantly raised to −143 kcal/mol from −355 kcal/mol and displayed the best potentiality. The interactions between S protein along with small molecule compounds and ACE2 are shown in Figure 6.

## 4. Discussion

SARS-CoV-2 continues to infect, hospitalize and kill millions of people worldwide (Drożdżal et al., 2021). The virus infects human ACE2 through the binding of the virus RBD of its S protein (Lan et al., 2020). SARS-CoV-2 Delta and Lambda variants are the worldwide epidemic virus strains before the prevalence of the Omicron variant. They have named a variety of concerns (VOC) and a variety of interest (VOI), respectively (Markarian et al., 2022). In this study, we constructed a protein model of the CTD of the S protein of SARS-CoV-2 Delta and Lambda variants and used molecular docking and MD simulation methods to study the infection mediated by all plant chemicals in the Chinese herbal medicine ingredient database (TCMSP) and SARS-CoV-2. The virus RBD of S protein is the region where SARS-CoV-2 binds to the human ACE2 receptor. The mutation of the RBD region makes SARS-CoV-2 Delta and Lambda variants more beneficial to the combination with ACE2 and immune escape (Arbeitman et al., 2021). Our research showed that the stable binding of four compounds (Casuarictin, Heterophyllin D, Protohypericin, and Glansin B) to the mutation sites of SARS-CoV-2 Delta and Lambda variants could weaken the stability of the binding of the RBD region of the virus to the receptor ACE2. Therefore, we speculate that these compounds (Casuarictin, Heterophyllin D, Protohypericin, and Glansin B) can weaken the spread of SARS-CoV-2 in the population by acting on the RBD domain of the virus.

S protein is a potential fragment that can be used as an antigen in vaccine development (Zhang et al., 2020). This protein plays a crucial role in the first step of the infection process because it binds to the ACE2 receptor and then enters the host cell (Petruk et al., 2020). According to the mutation sites of SARS-CoV-2 Delta and Lambda mutants, we constructed reasonable CTD protein models of the S protein. Delta strain was formed by mutating L452R and T478K in the RBD region of S protein, while the Lambda strain was formed by mutating L452Q and F490S in the RBD region (Hamill et al., 2022; Wang et al., 2022). In the constructed protein model, we studied the binding ability of 12,978 medicinal natural plant chemicals to SARS-CoV-2 S protein. In this study, more than 65% of the compounds in the molecular docking of Lambda and Delta mutants showed a good binding ability to their respective RBD regions (docking score ≤ −6), which were 65.61% (8,515/12,978) and 65.28% (8,472/12,978) respectively (Figure 2), indicating that Chinese herbal medicine is a huge database with great potential to find anti-SARS-CoV-2 compounds. The compounds clematichinenoside AR2 (−9.7), atratoglaucoside, b (−9.5), physalin b (−9.5), atratoglaucoside, a (−9.4), Ochnaflavone (−9.3),cyclopseudo-hypericin (−9.3), periplocoside J (−9.3), Bryonolic acid (−9.3), solamargine (−9.3), and salaspermic acid (−9.2) showed good binding ability with Lambda mutants, while the compounds neo-przewaquinone a (−10), Wikstrosin (−9.7), xilingsaponin A (−9.6), ardisianoside G (−9.6), 23-epi-26-deoxyactein (−9.6), mulberrofuran K (−9.6), Timosaponin A III (−9.5), physalin b (−9.5), 26-O-β-D-glucopyranosylnuatigenin-3-O-α-L-rhamnopyranosyl(1→2)-o-[β-D-glucopyranosyl(1→4)]-β-D-glucopyranoside (−9.5), and Ziebeimine (−9.4) showed good binding ability with Delta mutant (Tables 1, 2). Among the screened compounds with high scores, some have been reported to have antiviral activity, such as Hypericin, bryonolic acid, solamargine, and salaspermic acid, and some have anti-inflammatory effects, such as ochnaflavone, mulberrofuran K and timosaponin A-III. Hypericin is reported to have inhibition activity to α Coronavirus by targeting 3CL protease (Zhang et al., 2021). In addition, Hypericin can block the function of HSV-1 alkaline nuclease and inhibit virus replication (Cao et al., 2022). A study reported that bryonolic acid targeted the hotspot residues of SARS-CoV-2 main protease (Mpro and S protein), which has an essential role in mediating the viral replication therefore compounds targeting this key enzyme are expected to block the viral replication and transcription (Alagu Lakshmi et al., 2021). Chou et al. (2012) showed solamargine had potent activity against HBsAg, with an IC50 of 1.57 microM. Salaspermic acid inhibited HIV reverse transcriptase and HIV replication in H9 lymphocyte cells (Chen et al., 1992). Ochnaflavone, a double flavonoid compound, has the activity of resisting Escherichia coli, Staphylococcus aureus, Enterococcus faecalis, and Pseudomonas aeruginosa (Makhafola et al., 2012). Mulberrofuran K showed anti-inflammatory activities in lipopolysaccharide (LPS)-stimulated murine macrophages by inhibiting transcriptional activation of nuclear factor-κB (NF-κB) and extracellular-regulated kinases (ERK; Shim et al., 2018). Oral administration of timosaponin A-III at 25–50 mg/kg significantly inhibited the inflammatory markers in LPS-induced ALI mice, including the lung inflammatory index and the total number of inflammatory cells in the bronchoalveolar lavage fluid (BALF) (Park et al., 2018).

Among the top 100 compounds with good binding energy, we summarized the top 10 compounds that interact with mutant bases (Figures 3, 4, Tables 3, 4). All of these candidate ingredients showed a strong affinity for receptor molecular target sites with high binding energy. The receptor-ligand complex with the high score is stable through non-covalent interactions such as hydrogen bonding, van der Waals, and electrostatic interaction (Chen and Kurgan, 2009). These interactions are indeed prerequisites for biological functions and the success of drug development. Nine compounds (Casuarictin, C10230, Periplocoside J, Cyclopseudo-hypericin, Glaucoside, d, Hypericin, β1-solamargine, Mulberrofuran Q, and Atratoglaucoside, b) interacted with one mutation site of Lambda strain, and compound Heterophylliin D interacted with two mutation sites (Figure 3). In addition, compounds Glansrin B, Ardisiacrispin A, Periplocoside J, Cyclopseudo-hypericin, Amentoflavone, Ochnaflavone, Hypericin, Protohypericin, Datuarmeteloside B, and Primulanin interacted with a mutant base (L452R) of Delta strain, and compound Protohypericin also interacted with the mutant base T478K in another conformation (Figure 4). Casuarictin showed a robust binding activity with Lambda S-CTD by presenting a docking score of −9.1 and interacted with mutation residues (Q452) through conventional hydrogen bonding interaction (Figure 3A). In contrast, Heterophylliin D interacted with mutation residues (Q452) through conventional hydrogen bonding interaction and mutation residues (SERB490) through van der Waals bonding interaction (−8.1; Figure 3D). In addition, Glansrin B demonstrated vigorous binding activity with Delta S-CTD by exhibiting docking scores (−8.9), compared to Protohypericin, which presented a −8.2-docking score with Delta S-CTD and interaction with amino acid residue (R452) *via* conventional hydrogen bond interaction (Figures 4A, I).

To further observe the stability of the compounds binding to the mutant sites of Lambda and Delta strains, MD analysis was carried out for Casuarictin, Heterophylliin D, Protohypericin, and Glansrin B. RMSD is a parameter to calculate the distance between protein atoms. The average distance between atoms in target proteins that are unbound and ligand/standard inhibitor bound allows us to evaluate the comparative conformation and stability of proteins (Gupta et al., 2021). Casuarictin and Heterophylliin D bind to the S-CTD of Lambda strain converged at the beginning of the RMSD simulation and remained stable in the subsequent simulations (Figures 5A, B). And Protohypericin and Glansrin B bind to S-CTD of Delta strain also display reliable stability (Figures 5C, D). RMSF is an important parameter used to evaluate the changes of protein atoms in the whole time period from the reference position. This allows us to study the comparison results of target protein fractions (residues) before and after ligand binding. S-CTD of Delta strain and Lambda strain still had low RMSF fluctuations within 3 Å after Casuarictin and Heterophylliin D, or Protohypericin and Glansrin B binding, respectively, indicating that these proteins were low in flexibility and tightly bound to small molecules (Figures 5E–H). It is noteworthy that the compound casuarictin and the compound Protohypericin have been reported to have antiviral effects. Chandra et al. (2022) reported that casuarictin was identified to bind with the M Pro with the numerically lowest binding energies (−12.2 kcal/mol), with an alliance of five hydrogen bonds with amino acids T199 (3.02 Å), D197 (3.79 Å), R131 (2.76 Å), K137 (2.46 Å), and L287 (3.53 Å) and three hydrophobic interactions with amino acids L287 (5.30 Å), Leu272 (5.12 Å), and Y239 (5.20 Å). Tamura et al. (2010) used the model virus disclosed casuarictin as the HCV invasion inhibitor, which showed 87.4% inhibition rate and 45.1% inhibition rate to E1E2 virus and G* virus with a concentration of 10 μM, respectively. Casuarictin, which acts as a pure NF-κB inhibitor, inhibited IL-8 secretion in TNFα-treated human gastric epithelial cells by dampening the NF-κB signaling (Fumagalli et al., 2016). Protohypericin showed antiviral activity against a normal laboratory HCMV strain, AD-169, with an IC50 of 5.7 μM (Barnard et al., 1992). In addition, Protohypericin showed an anti-influenza virus activity (3.1 ng/mL) by the HA-assay (Yasuda et al., 2010).

The SARS-CoV-2 S protein, transmembrane protease serine 2 (TMPRSS2), and human receptor ACE2 are the main determinants of host pathogens affecting infection. The amino acid mutation of S protein, TMPRSS2, and ACE2 binding sites changed the protein affinity, which may affect the structural stability of the complex (Beacon et al., 2021; AlGhamdi et al., 2022). Therefore, S glycoprotein and host cell receptor ACE2 are one of the drug targets of SARS-CoV-2. To verify the binding complex of compounds (Casuarictin, Heterophyllin D, Protohypericin, and Glansin B) with S protein will affect the binding ability with human ACE2 receptor, the protein–protein docking method was used to observe the binding stability between small molecule-S protein complex and ACE2 receptor. Binding Energy (kcal/mol) was used to indicate the stability between two proteins. The lower the score, the stronger the bond. Our research indicated that the binding of four compounds (Casuarictin, Heterophyllin D, Protohypericin, and Glansin B) with S protein decreased the binding stability between S protein and ACE2 receptor, with the binding energy of small molecule-S protein complex to ACE2 increases (Table 7). It was worth noting that compared with the Lambda strain (−349 kcal/mol) and Delta strain (−355 kcal/mol), the binding energy of the standard strain and ACE2 (−330 kcal/mol) was higher. This result showed that the combination of the Lambda strain and Delta strain with ACE2 was strengthened, which also explained the high transmissibility of variant strains in clinical practice. Therefore, from this perspective, the combination of compounds (Casuarictin, Heterophyllin D, Protohypericin, and Glansin B) and S protein can reduce the strong infectivity of Lambda and Delta mutants to a certain extent.

## 5. Conclusion

This study explored the biological activities of 12,978 small molecules in Chinese herbal medicine through molecular docking and MD simulation analysis to prevent and treat COVID-19 infection, especially Lambda and Delta variants. In the docking results, more than 65% of the compounds had a relatively stable binding ability with S proteins. Among them, compounds Casuarictin, Heterophyllin D, Protohypericin, and Glansin B showed possible antagonistic resistance to the mutation sites of Lambda and Delta mutants and had significant binding energy. The MD simulation verified that these four phytochemicals, as strong interaction compounds, stabilized with the minimum deviation from the interaction site within the observed total simulation time. In addition, protein–protein docking between the complexes of these plant chemicals and S proteins and ACE2 receptors was carried out to evaluate their binding stability. The results showed that they could reduce the binding ability of S proteins to ACE2. Therefore, these phytochemicals may be feasible candidate drugs against SARS-CoV-2. However, extensive preclinical studies are needed to determine their effectiveness as antiviral agents.

## Data availability statement

The datasets presented in this study can be found in online repositories. The names of the repository/repositories and accession number(s) can be found in the article/Supplementary material.

## Author contributions

YL conceived and designed the study. TH conducted the molecular docking and simulation and drafted the manuscript. ZL conducted data analysis and plotted the figures. LJ and PW conducted data analysis and performed literature searches. GL, XL, and YL reviewed and revised the manuscript. All authors contributed to the article and approved the submitted version.

## Funding

This research was funded by the China Postdoctoral Science Foundation (grant no. 2021M700965), Guangdong Provincial Bureau of Traditional Chinese Medicine Research Foundation (grant no. 20231093), National Natural Science Foundation of China (grant no. 81973814), and Science, Technology and Innovation Commission of Shenzhen Municipality (grant no. JSGG20220226090550002).

## Conflict of interest

The authors declare that the research was conducted in the absence of any commercial or financial relationships that could be construed as a potential conflict of interest.

## Publisher’s note

All claims expressed in this article are solely those of the authors and do not necessarily represent those of their affiliated organizations, or those of the publisher, the editors and the reviewers. Any product that may be evaluated in this article, or claim that may be made by its manufacturer, is not guaranteed or endorsed by the publisher.

## Footnotes

^1^ https://www.rcsb.org/

^2^ https://saves.mbi.ucla.edu/

^3^ https://old.tcmsp-e.com/tcmsp.php

^4^ http://hdock.phys.hust.edu.cn/

## Abbreviations

ACE2: Angiotensin-converting enzyme 2
MD: Molecular dynamics
S: Spike
RBD: Receptor binding domain
VOC: Variety of concern
NAb: Neutralizing antibodies
TCMSP: Traditional Chinese Medicine Systems Pharmacology Database and Analysis Platform
Leu, L: leucine
Gln, Q: glutamine
F, F: Fnylalanine
Ser, S: serine
Arg, R: Arginine
Thr, T: Threonine
Lys, K: Lysine
CTD: C-terminal domain
RMSD: Root Mean Square Deviation
RMSF: Root Mean Square Fluctuation
VOI: Variety of interest
