# Supplementary figures and images for: Identification of natural compounds as SARS-CoV-2 inhibitors via molecular docking and molecular dynamic simulation
Source: Front Microbiol. 2023 Feb 1;13:1095068. doi: 10.3389/fmicb.2022.1095068 (PMC9930647; doi:10.3389/fmicb.2022.1095068)

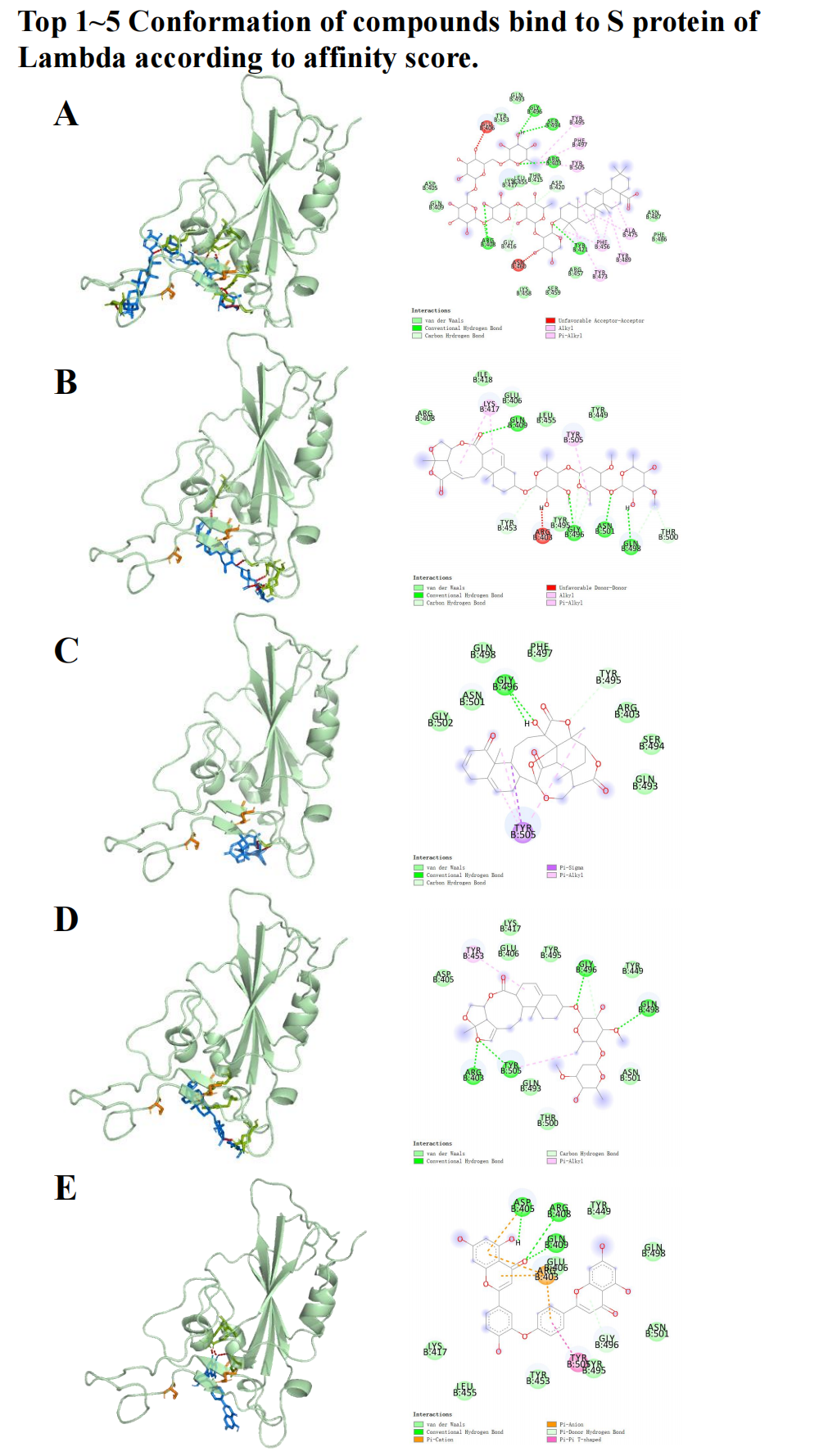

Supplement: Supplementary file 1 [file Image_1.TIF]

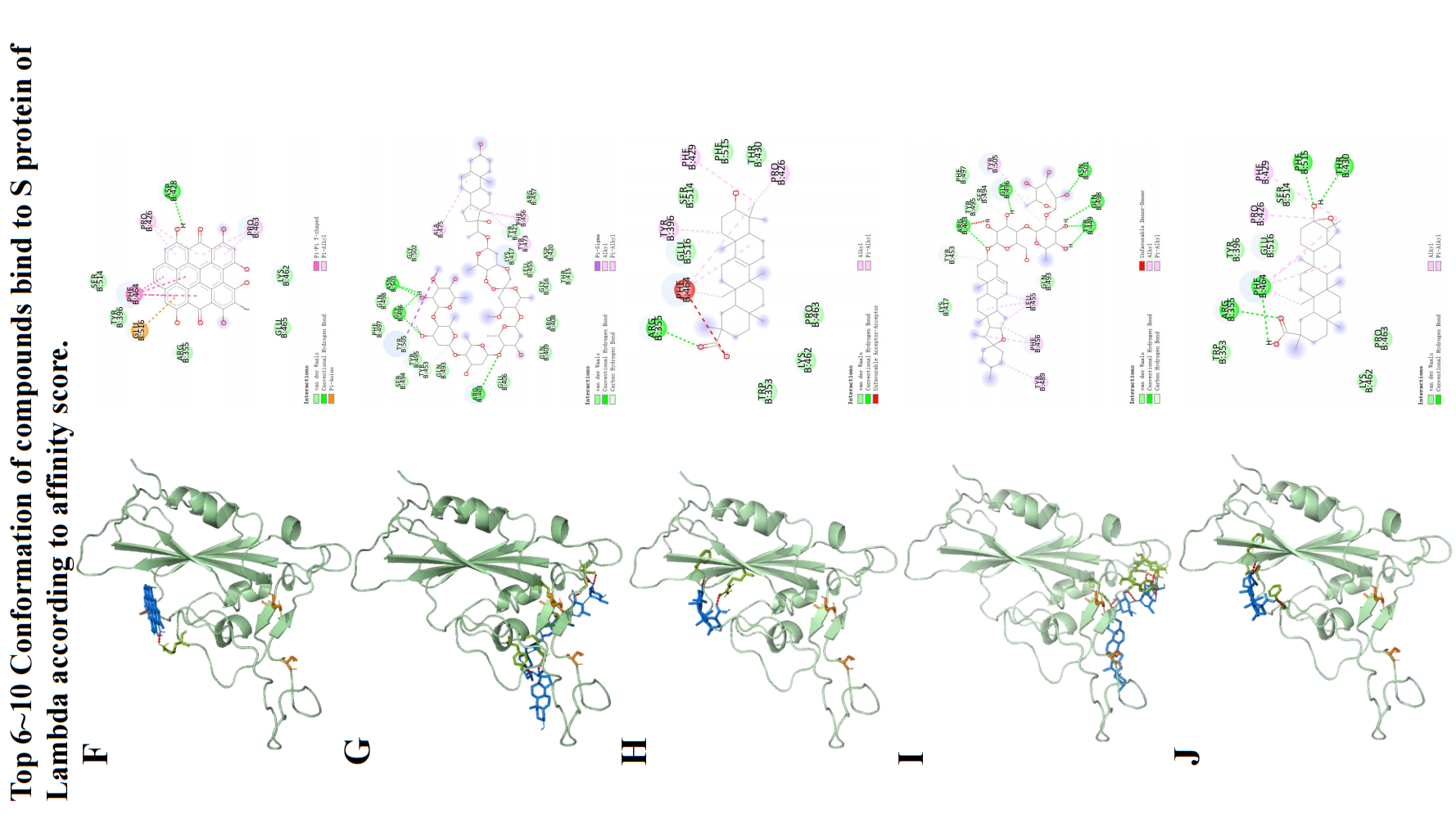

Supplement: Supplementary file 2 [file Image_2.TIF]

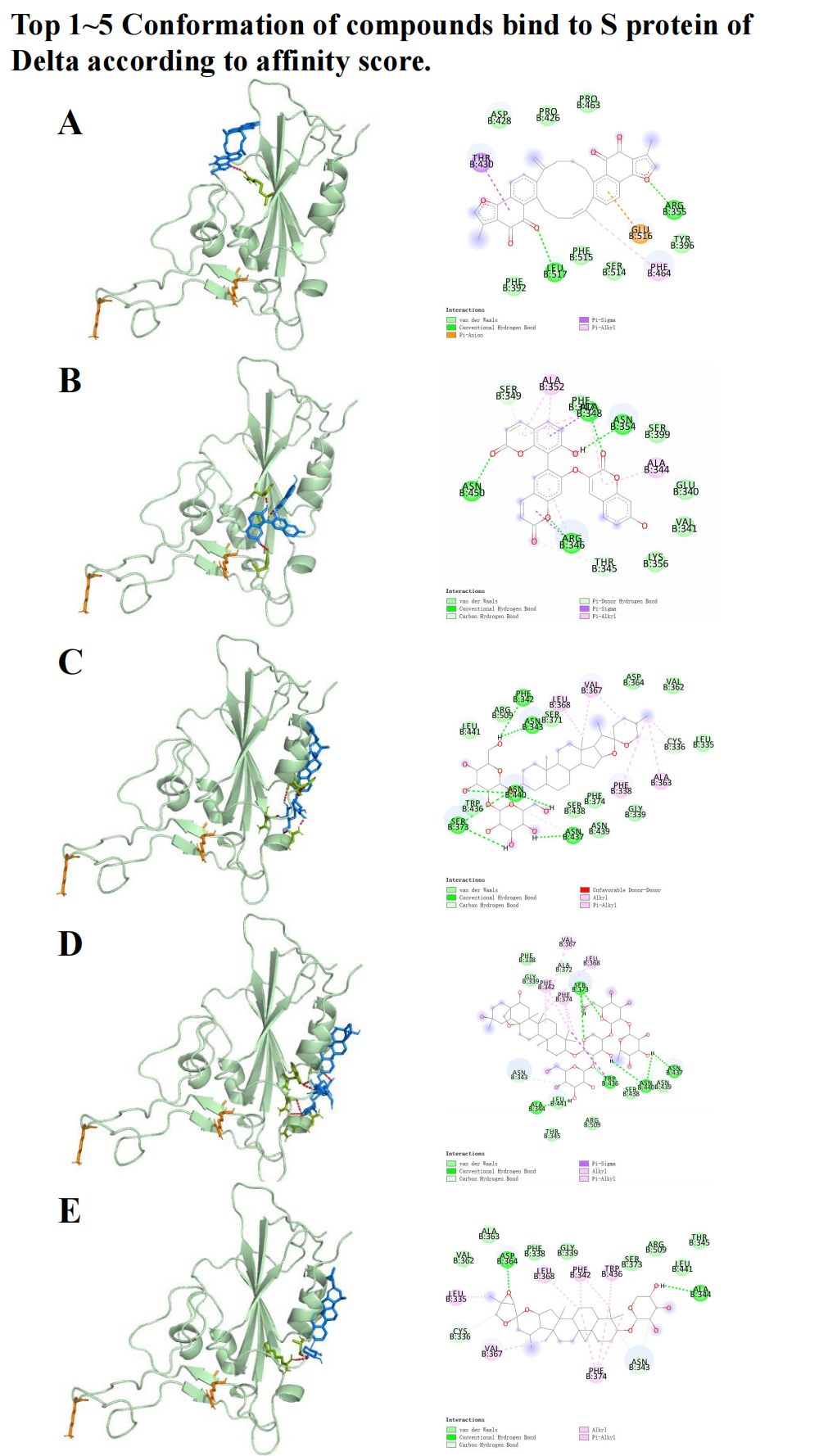

Supplement: Supplementary file 3 [file Image_3.TIF]

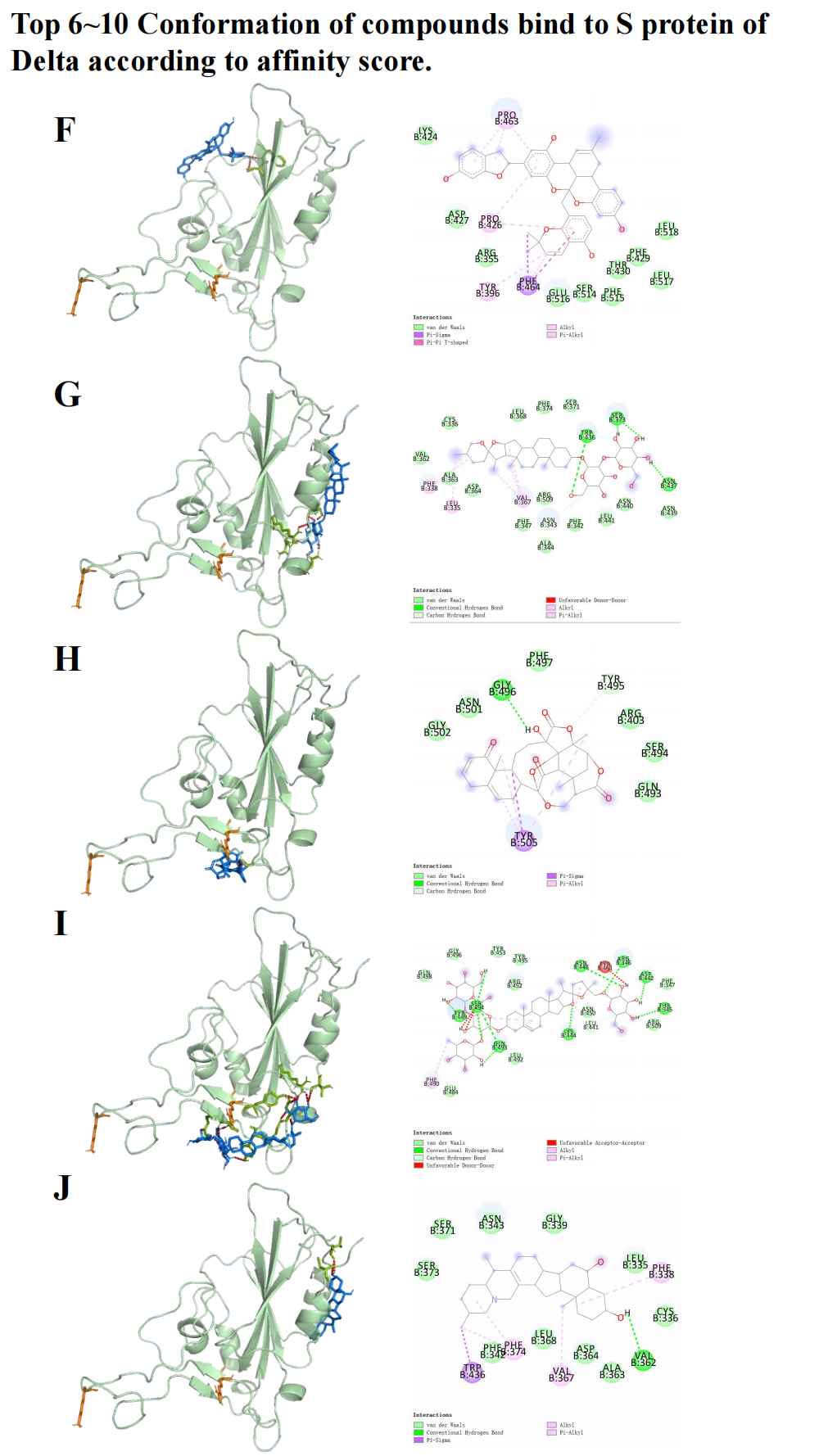

Supplement: Supplementary file 4 [file Image_4.TIF]
